# The epithelial polarity genes *frazzled* and *GUK-holder* adjust morphogen gradients to coordinate changes in cell position with cell fate specification

**DOI:** 10.1371/journal.pbio.3002021

**Published:** 2023-03-13

**Authors:** Yongqiang Xue, Aravindan Krishnan, Juan Sebastian Chahda, Robert Allen Schweickart, Rui Sousa-Neves, Claudia Mieko Mizutani

**Affiliations:** 1 Department of Biology, Case Western Reserve University, Cleveland, Ohio, United States of America; 2 Department of Genetics and Genome Sciences, Case Western Reserve University, Cleveland, Ohio, United States of America; University of Zurich, SWITZERLAND

## Abstract

Morphogenetic gradients specify distinct cell populations within tissues. Originally, morphogens were conceived as substances that act on a static field of cells, yet cells usually move during development. Thus, the way cell fates are defined in moving cells remains a significant and largely unsolved problem. Here, we investigated this issue using spatial referencing of cells and 3D spatial statistics in the *Drosophila* blastoderm to reveal how cell density responds to morphogenetic activity. We show that the morphogen decapentaplegic (DPP) attracts cells towards its peak levels in the dorsal midline, whereas dorsal (DL) stalls them ventrally. We identified *frazzled* and *GUK-holder* as the downstream effectors regulated by these morphogens that constrict cells and provide the mechanical force necessary to draw cells dorsally. Surprisingly, GUKH and FRA modulate the DL and DPP gradient levels and this regulation creates a very precise mechanism of coordinating cell movement and fate specification.

## Introduction

The “French Flag model” of positional information is a central model in developmental biology that explains how cell fates are specified within broad regions of developing embryos, limbs, and other organs. In this model, cells are thought to interpret threshold levels of a morphogen according to their spatial position and acquire distinct fates through the activation of specific gene expression programs [1–4]. Despite its immense power to explain diverse phenomena such as embryonic axial patterning and segregation of different neuronal populations in the nervous system, a major simplification is frequently overlooked in this model. Namely, it is assumed that morphogen thresholds reach and modify a static cell population. However, this condition is seldom if ever met in most developmental contexts, which generally involve a dynamic displacement of cells at the same time they read the instructive thresholds that determine their fates. Here, we sought to understand how tissues coordinate cell movements with cell fate specification during pattern formation.

**Fig 1:**
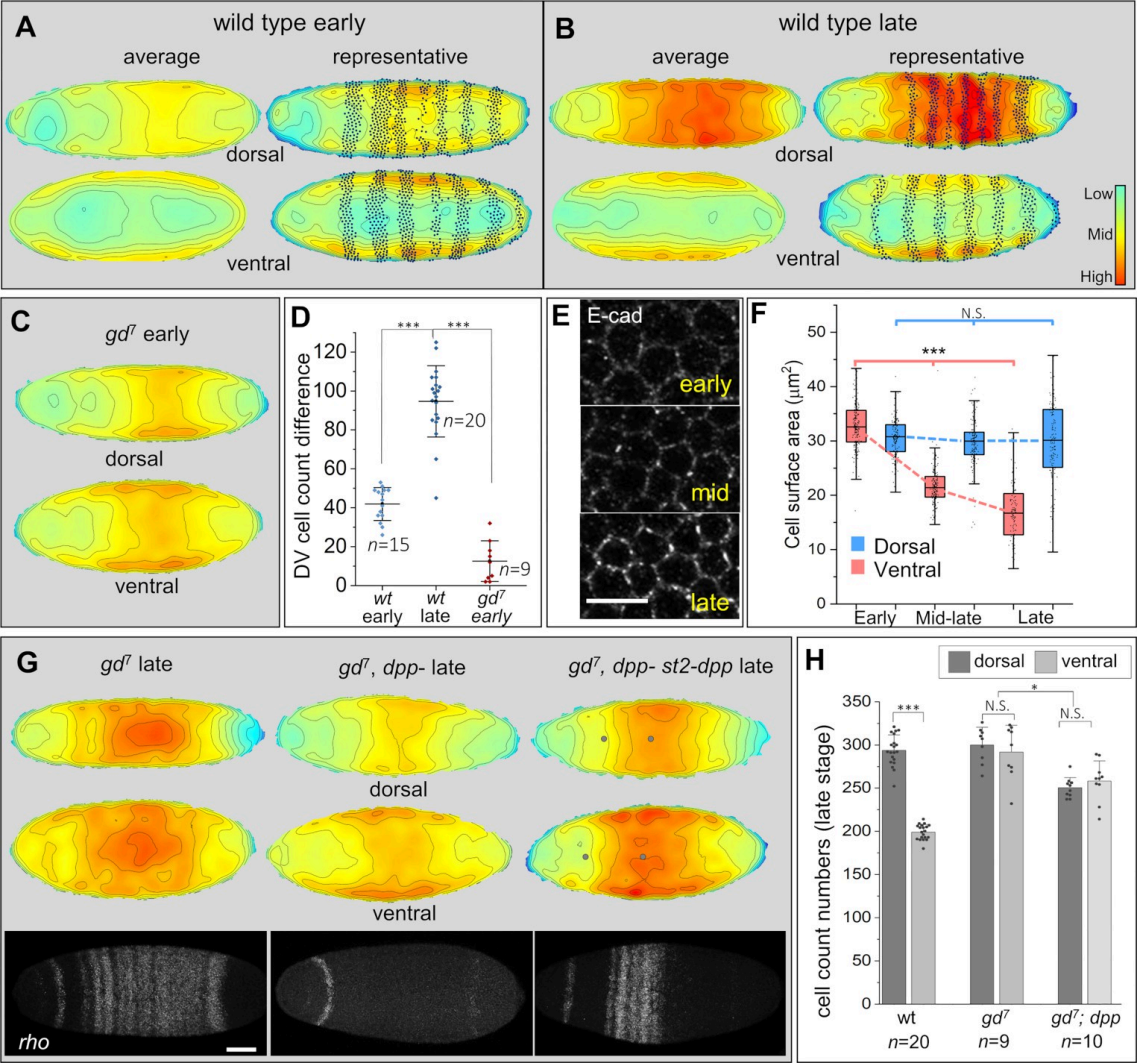
DPP is the morphogen that attracts cells dorsally. (A, B) Cell density heatmaps of dorsal and ventral sides of wild-type embryos at early (A) and late (B) cellularization stages. “Average heatmaps” combine data of 9 embryos for all genotypes, except for *gd*^*7*^;*dpp-* (*n* = 5). “Representative heatmap” shows a single embryo with A/P marker *ftz* (black dots). Note the higher dorsal cell density in early stage that increases in late stage. (C) *gd*^*7*^ mutants with no DL gradient loose early asymmetry of cell densities. (D) Dorsal and ventral cell count differences of embryos shown in (A–C). (E) anti-E-CAD membrane labeling of dorsal cells in wild type during cellularization. (F) Quantification of cell constriction over time. Dorsal cells show significant decrease in segmented surface area from early to mid- to late stages, while ventral cells of same embryos do not. (G, H) DPP increases cell density in the absence of DL. (G) Average cell density heatmaps in late stage *gd*^*7*^ embryos without DL gradient (top left). The high-density levels in ventral and dorsal surfaces is abolished when DPP is also removed (*gd*^*7*^;*dpp-* embryos, top center) and restored by the ectopic expression of *dpp* (*gd*^*7*^*;dpp- st2-dpp* embryos, top right; dots indicate margins of *rho* domain). Changes in the expression of DPP-target *rho* in these genetic backgrounds (bottom) confirm the manipulations. (H) Dorsal and ventral cell counts in wild type, *gd*^*7*^ and *gd*^*7*^*;dpp-* embryos at late cellularization stage. Error bars, standard deviation. N.S., not significant. Asterisks, threshold values based on *p*-values calculated with two-tail Mann–Whitney test (D, F, H, comparison of different genotypes and stages) or two-tailed Wilcoxon signed-rank test (H, comparison of D/V surfaces of same genotypes and stages). *p < 0.05, ***p < 0.0001. Scale bars, 10 μm (E) and 60 μm (G). Metadata for the graphs shown in D, F, and H can be found at Supporting information S1 Metadata. A/P, antero-posterior; DL, dorsal; DPP, decapentaplegic; D/V, dorso-ventral; FTZ, fushi tarazu; GD, gastrulation defective; ST2, even skipped stripe 2.

To that end, we analyzed the *Drosophila* blastoderm, a phase of embryonic development in which the syncytium nuclei behave as compartmentalized open cells [5–8] that move in a single plane on the surface of embryos as they acquire distinct fates. For a long time, cells at this embryonic stage were treated as a static field. However, this view changed with the discovery that not only cells move but they actually follow stereotyped trajectories that ultimately establish well-defined patterns of cell densities along the antero-posterior (A/P) and dorso-ventral (D/V) axes [9–11] (Fig 1). In particular, cells from the lateral sides of the embryo and from the anterior and posterior poles move towards the dorsal midline, whereas cells within the ventral region remain mostly immotile with only slight movements towards the posterior region. Noteworthy, these stereotyped cell movements are disrupted in mutant embryos without either bicoid (BCD) or dorsal/NFκB (DL/NFκ-B) gradients, indicating that they are regulated by morphogens that pattern the A/P and D/V embryonic axes, respectively [9]. Additional evidence of a genetic control of these movements comes from the fact that different lineages of Drosophilids display species-specific patterns of cell density [12].

The discovery of global stereotyped cell movements in the blastoderm opens the possibility of investigating the mechanisms by which morphogens coordinate cell fate specification relative to cell position. This exquisitely simple, bidimensional embryonic patterning allows for referencing each cell in space and in this way to ask how morphogens control the stereotyped cell trajectories. In addition, this model allows for testing possible molecular effectors that coordinate this process. To address these issues, we focused on the stereotyped cell movements along the D/V axis, where the mesoderm, neuroectoderm, and ectoderm are formed. The formation of these domains depends on a ventral-to-dorsal nuclear gradient level of the transcription factor DL/NFκ-B and an opposing dorsal-to-ventral gradient of secreted decapentaplegic/bone morphogenetic 4 (DPP/BMP-4) [13–19]. DL activates mesodermal genes such as *snail* (*sna*) and neuroectodermal genes, such as *short gastrulation* (*sog*) and *intermediate nervous system defective* (*ind*), and represses ectodermal genes, such as *dpp*. The DL gradient is established by a maternal signaling pathway that regulates the graded activation of the Toll receptor by Spätzle ligand along the DV axis leading to the selective transport of DL into the nucleus. DPP activates ectodermal genes, such as *rhomboid* (*rho*) and *race* and represses neuroectodermal genes, such as *muscle segment homeotic* (*msh*) and *ind*. The DPP gradient is established zygotically and depends on the interaction of various extracellular modulators. Here, we show that DPP, and not DL, directly attracts cells to the dorsal region and that DL stalls cells ventrally by excluding DPP expression. Through an in silico screen, we identified *GUK-holder (gukh)* and *frazzled (fra)* as the effector genes that respond to DPP and/or DL and control the stereotyped cell movements. We show that both genes are required for the correct formation of cell density patterns and the specification of the mesodermal, neuroectodermal, and ectodermal expression domains. Finally, we show that *fra* and *gukh* modulate the shapes of the gradients of DL and DPP. Together, our results provide evidence that DPP instructs cells about their fate in a dosage-dependent manner and adjusts these thresholds by positioning cells in space.

## Results

### Cells move towards the dorsal midline during cellularization in a stereotyped fashion

We began our study by first analyzing the formation of cell density patterns resulting from cell movements during the blastoderm stage. To quantify these patterns along the D/V axis, we analyzed ventral and dorsal halves of individual embryos precisely oriented in each position according to D/V and A/P markers (*rhomboid* (*rho*), *snail* (*sna*), *intermediate neuroblasts defective (ind)*, *even-skipped* (*eve*), and *fushi-tarazu (ftz)* (see Methods). We focused on early and late cellularization stages defined by initial and fully extended membrane invagination on the ventral side, respectively. Images obtained from these stages were then segmented to obtain the centroids of each nucleus, and the 3D cell features were imported into geographic information system (GIS) to generate average cell density heatmaps for the dorsal and ventral regions and to analyze the data with spatial statistics. In addition to heatmaps and hot/cold spot maps, we also tested for differences in cell count numbers (see Methods).

Our results show that at the cellularization onset, there is already a slightly higher number of dorsal cells than ventral cells (Fig 1A). These differences are established prior to cellularization by the DL gradient as evidenced by the fact that embryos without nuclear DL have equal cell densities in the ventral and dorsal sides at the beginning of cellularization (Fig 1C and 1D). In agreement with previous reports [9,12], by the end of cellularization, the asymmetric pattern of high density of cells within the dorsal region versus low density in the ventral region becomes evident in density heatmaps of late stage embryos (Fig 1B and 1D).

Since the total cell number in the embryo remains constant during cellularization [20,21] and apoptosis is absent during this stage [22], the emergence of a dorsal region of high cell density reflects the movement of cells towards the dorsal midline [9]. To visualize these movements, we analyzed time-lapse videos of live embryos expressing the cell membrane protein E-Cadherin/Shotgun-GFP (E-CAD/Shg-GFP) [23] as well as E-CAD in fixed embryos throughout cellularization (Fig 1D). The data obtained with E-CAD-GFP agree with previous analyses using Histone-GFP [9], but since E-CAD-GFP labels cell contours, our analyses rule out the possibility of nuclear movement within cells and reveal that the cell movements involve cell constriction with no evident intercalation between cells (S1 Video). We measured the segmented surface areas of individual dorsal cells expressing E-CAD-GFP in time-lapse images (S2 Video and S1 Fig) and from dorsal and ventral cells of same fixed embryos at 3 distinct stages (Fig 1E and 1F). These results show that the apical surface of dorsal cells becomes constricted and tightened as cells move dorsally, resulting in a significant decrease in cell size from early to mid and to late stage. In contrast, cells located in the ventral side of the embryo do not constrict over time (Fig 1F). Thus, these experiments confirm the stereotyped cell movements from the lateral regions and poles towards the dorsal midline center, resulting in an increase in cell density on the dorsal surface of the embryo compared to the ventral surface.

### DL regulates cell movements indirectly through DPP

From the data presented above and previous reports, it is clear that cells are attracted to the dorsal region and their density increases in this region over time. To a large extent, this pattern of cell clustering is the mirror image of the DL gradient, which decreases continuously towards the dorsal side. In addition, the asymmetric dorsal clustering of cells was shown to be abrogated in embryos with no nuclear DL [9]. However, if DL regulated this dorsal-bound cell movement directly, then this would imply that it does so by creating cell repulsion. In this case, we should expect repulsion to reach its maximum in the ventral region where the levels of DL peak. Nevertheless, in this region, the cell movement is virtually inexistent [9]. Thus, DL cannot be the morphogen that directly governs these cell movements, but rather it must do so indirectly through repression of another morphogen that attracts cells dorsally. The natural candidate to exert this activity is DPP, which meets the requirements of being repressed ventrally by DL and achieving peak levels in the dorsal midline, the site where cells are attracted to.

To understand the relationship between cell clustering and the DL and DPP gradients, we tested the effects caused by the removal of DL and DPP individually and simultaneously. First, we analyzed embryos without the DL gradient using the maternal mutation *gastrulation defective* (*gd*), which prevents the processing of Spätzle and the activation of Toll. In those embryos, the DL gradient is not formed because DL is absent from the nuclei. In addition, the DPP gradient is not formed because the relief of DL repression allows DPP to expand across the DV axis as can be seen by the ubiquitous activation of its target *rho* (Fig 1G). As expected, we observe a high cellular density across the D/V axis within the center region of these embryos (Fig 1B, 1G and 1H) [9]. The anterior and posterior poles have a low density as in the wild type, indicating that the movements controlled by the A/P coordinates are maintained, but these anterior/posterior-most cells move towards the center of the embryo without noticeable dorso-ventral differences (i.e., the directionality to the dorsal midline is lost in *gd* embryos). Next, to test if this cellular density packing stems from the indirect ubiquitous activation of DPP, we removed DPP from embryos without DL gradient (note loss of *rho* activation in Fig 1G). In contrast to the loss of DL only, embryos without DL and DPP gradients have a much lower cell density across the D/V axis (Fig 1G and 1H). Thus, DPP is required for cell clustering. To further test the ability of DPP to attract cells, we analyzed embryos without DL and DPP gradients expressing DPP orthogonally by using the *even-skipped stripe 2-dpp* (*st2-dpp*) construct [24]. These experiments show that DPP expressed in this position activates ectopically its target gene *rho* and indeed attracts cells to the center of the embryo across its entire circumference (Fig 1G and 1H).

**Fig 2:**
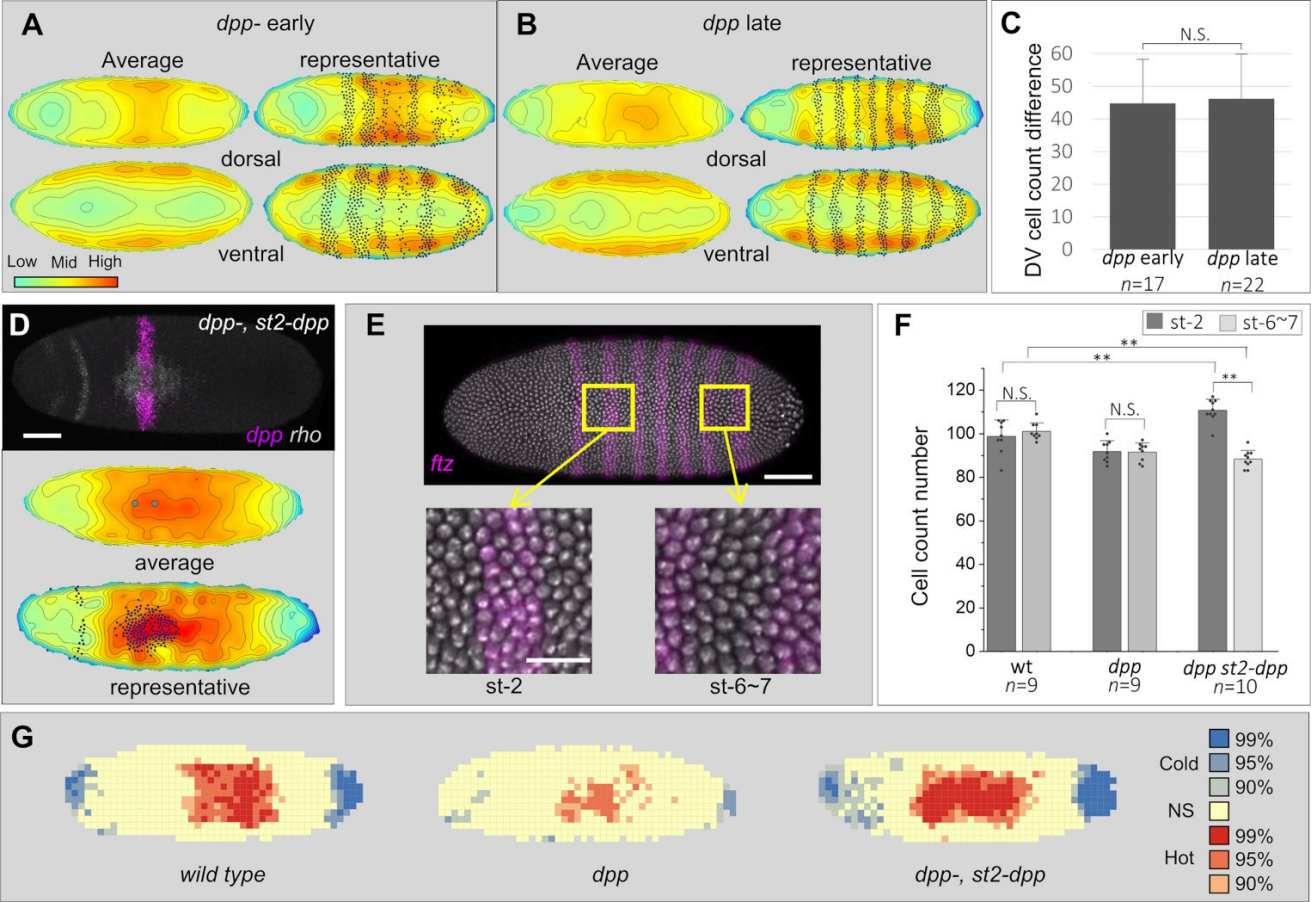
DPP pulls cells over long distances. Cell density heatmaps of *dpp-* embryos at early (A) and late (B) cellularization stages. The dorsal surface has slightly higher density than ventral surface that does not change over time. Average heatmap shown on left (*n* = 9). Representative heatmap on right shows *ftz* stripes (black dots). (C) Cell number differences between ventral and dorsal sides in *dpp-* at early and late stages. (D) Cell density heatmaps of the dorsal surface of late stage *dpp-* embryos ectopically expressing *dpp* (*st2-dpp*) (gray dots, stripe position). Black dots in representative heatmap show *rho*-expressing cells. (E) Selected regions for cell counts using *ftz* as an A/P landmark for nearby (st-2) and distant (st-6~7) regions from ectopic *dpp*. (F) Cell counts in late-stage wild type and *dpp-* and *dpp-*, *st2-dpp* embryos. Note that *st2-dpp* expression increases the density of cells in *ftz* stripe 2 and reduces the density of cells between stripes 6 and 7 compared to wild type. Error bars, standard deviation. N.S., not significant. Asterisks, threshold values based on *p*-values (**p < 0.01) calculated with two-tail Mann–Whitney test (different stages in C; genotype comparisons in F) or two-tailed Wilcoxon signed-rank test (st-2 and st-6~7 comparisons of same genotypes in F). (G) Hot spot analysis of dorsal surface of wild type, *dpp* and *dpp*, *st2-dpp* embryos. Legend on left side indicates color-code for confidence intervals of hot spots (red shades) and cold spots (blue shades), and nonsignificant regions (NS, yellow). Scale bars, 60 μm (D and E, top) and 20 μm (E, bottom). Metadata for the graphs shown in C and F can be found at Supporting information S1 Metadata. A/P, antero-posterior; DPP, decapentaplegic; FTZ, fushi tarazu; GD, gastrulation defective; ST2, even skipped stripe 2.

Several other pieces of evidence unambiguously demonstrate that DPP attracts cells. First, the cell density in the dorsal region never increases over time in embryos without DPP (Fig 2A–2C). Second, this phenotype can be rescued by the addition of *st2-dpp*. The rescued embryos have a broad area of high cell density on the dorsal surface beyond the sites of *dpp* RNA expression and *rho* activation (Fig 2D). Third, cell counts along the A/P axis of these embryos show a higher cell density anteriorly than posteriorly, showing a reorganization in the cell density along the A/P axis in response to the localized ectopic DPP source (Fig 2E and 2F). Finally, Getis-Ord Gi* spatial statistics show that while wild-type embryos have large hot spots in the central dorsal region flanked by cold spots in the poles (Fig 2G), the hot and cold spots are smaller in *dpp* embryos and there is an increase in randomly distributed cells (Fig 2G). The expression of *st2-dpp* reverts this phenotype similar to the wild type, with hot spots shifted more anteriorly (Fig 2G).

### A genome-wide search identifies *GUK*-*holder* and *frazzled* as candidate genes to regulate cell movements

The results above show that DPP attracts cells dorsally and DL stalls them ventrally by excluding DPP expression. However, since DL encodes a transcription factor and DPP encodes a secreted signaling protein, these cell movements must be enabled by downstream genes. To identify these effectors, we searched for genes likely to respond to DL and/or DPP gradients and that encode proteins with a role consistent with the regulation of cell movement. We screened the *Drosophila* genome for genes with similar developmental expression patterns to the DPP receptor thickveins using the existing modENCODE mRNAseq development database. Out of 101 genes identified, we selected those with predicted functions in cell migration, cell adhesion, and/or cytoskeleton regulation, as well as asymmetric expression along the D/V axis (see Methods for details).

This search led to the identification of 2 genes, *frazzled* (*fra*) and *GUK-holder* (*gukh*). Both genes have been implicated in migration in various developmental contexts but were not previously associated to either DPP or DL, and their early embryonic functions are unknown. *fra* is the *Drosophila* homolog of *Deleted in Colorectal Cancer* gene (*DCC*) and encodes a protein belonging to the immunoglobulin subfamily that functions as the receptor of Netrin [25]. FRA/DCC was previously implicated in glial and axonal migration, and migration of various cell types including cardiac, salivary gland, and mesenchymal cells in *Drosophila* [25–30]. *gukh* encodes a protein with a SCAR-WAVE domain predicted to act on the nucleation of actin filaments [31]. In addition, GUKH physically interacts with membrane-associated guanylate kinases (MAGUKs), such as DISCS LARGE (DLG) [31–33] and is required for the correct subcellular localization of the planar cell polarity proteins DLG and Scribble (SCRIB) [31,34]. In *Danio rerio*, its ortholog has been shown to regulate cell migration during craniofacial development [35]. Furthermore, the human orthologue of GUKH, the Nance–Horan syndrome gene (NHS), nucleates actin filaments [36]. Noteworthy, emerging work link both *fra* and *gukh* to epithelial polarity functions, morphogenesis, and regulation of adherens junctions along the cell apico-basal axis [29,34,37–39].

### DPP and DL regulate *gukh* expression and DL regulates *fra* expression

**Fig 3:**
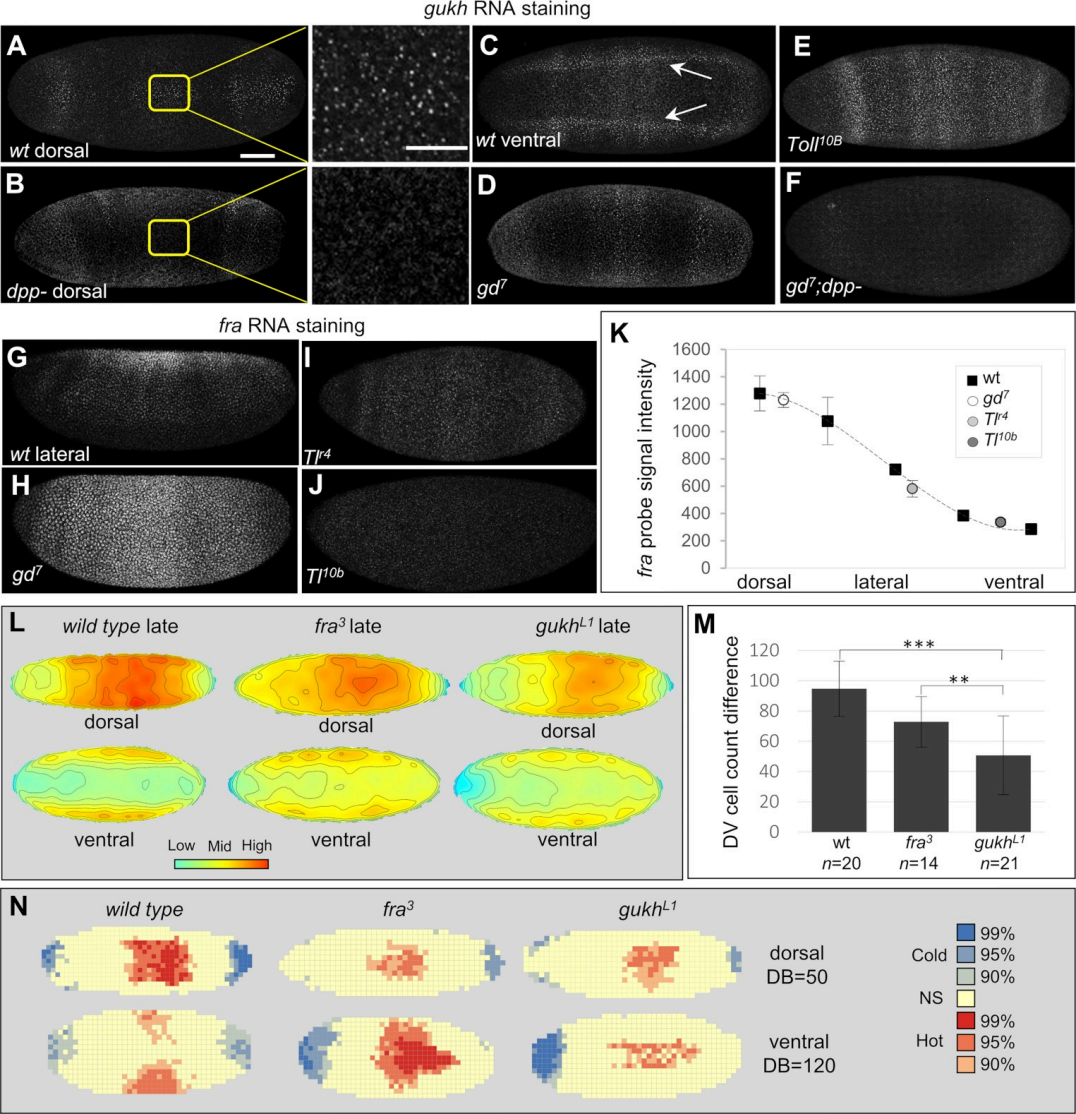
GUKH and FRA are DL and/or DPP targets that regulate coordinated cell movements. (A–F) *gukh* is activated by DPP and DL. In the wild type (wt), *gukh* RNA appears in a thin stripe in the dorsal midline (A) and a broad ventral domain with high levels in the ventral neuroectoderm (C, arrows; S1 Fig). (B) *gukh* dorsal expression is lost in *dpp-* embryo; compare nascent transcripts in insets in A, B (see also S1 Fig). (D) *gd*^*7*^ mutant without DL and ubiquitous DPP have expanded *gukh* dorsal domain across the D/V axis and loss of neuroectodermal expression domain. (E) *Toll*^*10b*^ mutant with ubiquitous high nuclear DL levels have *gukh* ventral expression domain expanded. (F) *gukh* expression is completely lost in the absence of DL and DPP in *gd*^*7*^*; dpp-*. (G) *fra* RNA forms a D/V gradient. Graded DL levels repress *fra* as seen by *fra* expansion in *gd*^*7*^ mutant with no DL (H) and increasingly lower levels in *Tl*^*r4*^ (I) and *Tl*^*10b*^ (J) mutants with intermediate and high nuclear DL levels (see also S1 Fig). (K) *fra* RNA levels decrease with increasing levels of DL in wild type and in mutants. (L) Average cell density heatmaps of late-stage wild type, *fra*^*3*^ and *gukh*^*L1*^ embryos (*n* = 9) show that the high cellular density in the dorsal side requires *fra* and *gukh*. Note also the higher ventral cellular density in the mutants compared to wild type (see also S2 Fig). (M) D/V cell count differences of genotypes in (L). Error bars, standard deviation. Asterisks, threshold values based on *p*-values calculated with two-tail Mann–Whitney test (**p < 0.001 and ***p < 0.0001). (N) Hot spot analysis of dorsal and ventral embryo surfaces of wild type, *fra* and *gukh*. Legend on left shows color-codes for confidence intervals; NS, not significant; DB, distance band value employed for each surface analyzed. Scale bars shown in A, 60 μm (whole mounts), 20 μm (inset). Metadata for the graphs shown in K and M can be found at Supporting information S1 Metadata. DL, dorsal; DPP, decapentaplegic; D/V, dorso-ventral FRA, frazzled; GD, gastrulation defective; GUKH, guk-holder.

We next tested if *gukh* and *fra* are regulated by DL and/or DPP. *gukh* RNA is strongly expressed in 2 lateral stripes in the ventral neuroectoderm, in the mesoderm, and in a thin stripe within the dorsal midline as revealed by sensitive fluorescent in situ hybridization (Figs 3A, 3C, S2A and S2B). Our results show that both DPP and DL are required to activate *gukh* in the dorsal and ventral regions, respectively. This regulation is evident in *dpp-* mutants where the expression of *gukh* in the dorsal midline is lost (Fig 3A and 3B; note the presence and absence of *gukh* nascent transcripts in high magnification boxes) and restored by the expression of *st2-dpp* (S2C and S2D Fig). Furthermore, in *gd*^*7*^ mutant embryos that cause the loss of nuclear DL and the activation of DPP signaling, the dorsal stripe of *gukh* expression expands throughout the embryo circumference (Fig 3D). Evidence that *gukh* is also activated by DL was obtained using *Tl*^*r4*^, a mutation that maintains uniform levels of nuclear DL around the entire embryo circumference [16,40]. In these embryos, the *gukh* ventral expression domain is expanded around their entire circumference (Fig 3E). Finally, *gukh* expression is completely lost in *gd*^*7*^, *dpp-* embryos that lack both DL and DPP (Fig 3F).

In contrast to the expression of GUKH that appears in discrete positions, *fra* RNAs are distributed in a dorsal to ventral gradient (Fig 3G) that is not regulated by DPP (S2E–S2H Fig) but generated by a dosage-dependent repression by DL. This can be best demonstrated by quantifying the levels of *fra* in embryos without nuclear DL (*gd*^*7*^ embryos) (Fig 3H) or intermediate (Fig 3I) and high levels of nuclear DL (Fig 3J) (i.e., using moderate and strong dominant mutations of *Tl*). Indeed, the intensity levels of *fra* RNA obtained in mutants that do not express DL or express DL uniformly at a single level, reproduce discrete points of the curve of decaying *fra* levels from dorsal to ventral regions in normal embryos (Fig 3K).

### *fra* and *gukh* are required for stereotyped cell movements

We next asked if the response of *fra* and *gukh* to the D/V gradients of DL and/or DPP is required for the stereotyped cell movements in the embryo. To address this issue, we analyzed cell density heatmaps and hot/cold spots of the null mutants *fra*^3^ and *gukh*^*L1*^. The heatmaps show that the cell density decreases in the dorsal region of mutant embryos and increases in the ventral region (Fig 3L). These findings are confirmed by direct cell counts that yield a significantly lower D/V cell count differences in *fra* and *gukh* embryos compared to the wild type (Fig 3M). The hot spot analyses in the dorsal region can still identify hot spot clusters in the mutants, and those are established with less cell numbers (Fig 3N). Noteworthy, we observe the emergence of cell density hot spots within the ventral midline of mutant embryos (Fig 3N). This finding contrasts to wild-type embryos, which typically show random cell distribution in the ventral region, and occasionally have few hot spots in more lateral regions but not in the ventral midline (Fig 3N). Finally, density heatmaps in the lateral region of the embryo corresponding to the neuroectoderm show a higher cell density in *fra*^*3*^ and *gukh*^*L1*^ than the wild type (S3 Fig), in agreement with a decreased attraction of lateral cells to the dorsal region. Taken together, these results show that *fra* and *gukh* are required for the stereotyped cell movements in the blastoderm.

### The cell movement towards the dorsal side of embryos deploys changes in cell area that are regulated by FRA and GUKH

**Fig 4:**
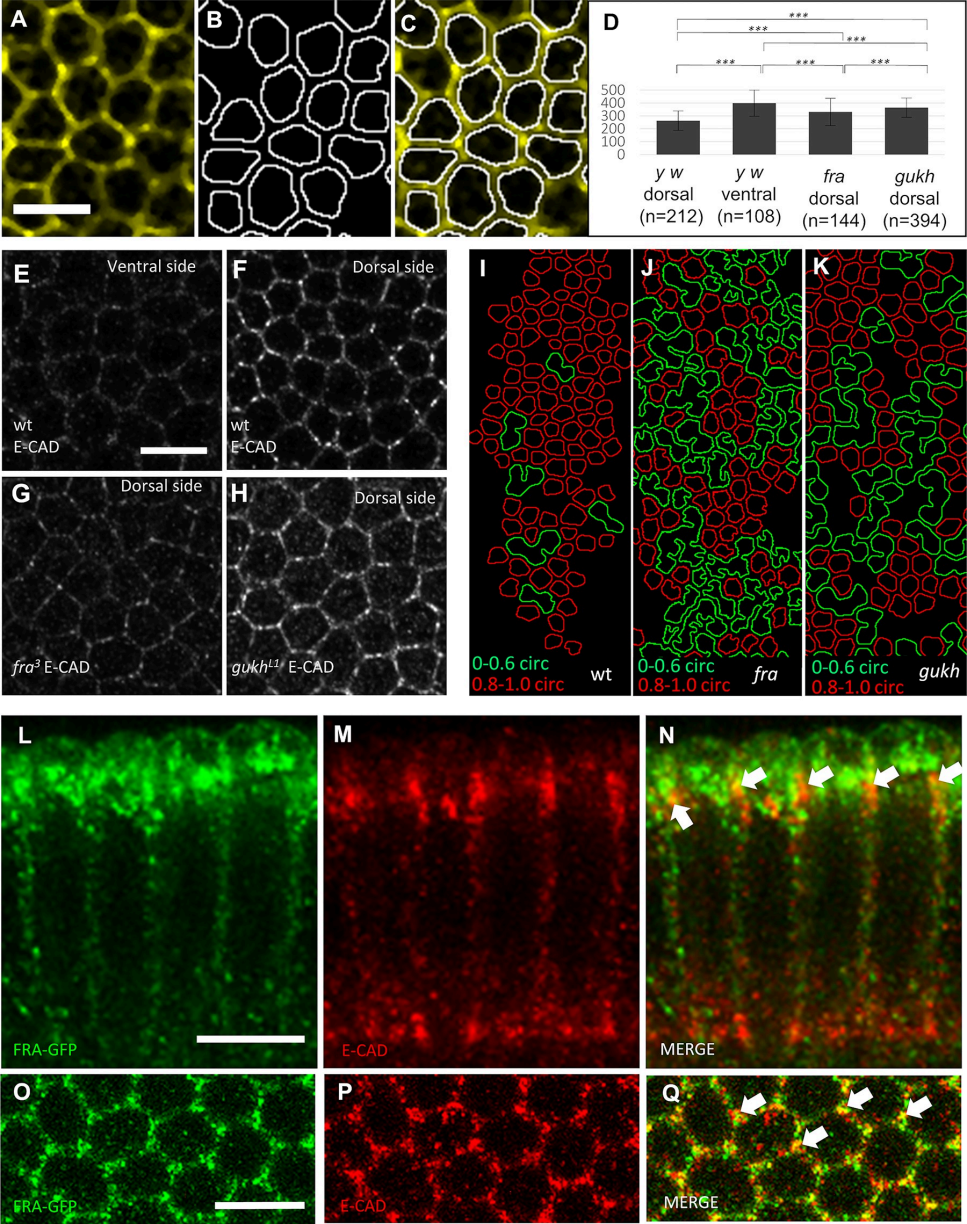
FRA and GUKH are required for cell constriction through apical localization of E-CAD at cell membranes. (A–C) Image segmentation for cell surface measurements. (A) Filtered image of anti-E-CAD staining. (B) Image segmentation shows outlines of membranes bordering E-CAD. (C) Merge of A, B. (D) Quantifications of surface areas from wild-type ventral and dorsal cells, and dorsal cells from *fra* and *gukh* mutant at late cellularization stage. In the wild type, dorsal cells are significantly smaller than ventral cells. In *fra* and *gukh*, dorsal cells are not constricted as dorsal cells in wild type. Error bars, standard deviation; *p*-values calculated with two-tail Mann–Whitney test (***p < 0.001). (E, F) Late-stage wild-type embryo stained for anti-E-CAD showing lower levels in ventral (E) than dorsal cells (F). Dorsal cells from *fra* (G) and *gukh* (H) stained for E-CAD. Note larger cell sizes with diffuse E-CAD signal compared to wild type, and low E-CAD membrane signal in *fra* (see also S3 Fig). (I–K) Segmented dorsal cells from wild type, *fra* and *gukh* stained with E-CAD. The low levels of E-CAD in the mutants result in less cells with high circularity (red outlines) and more cells with lower circularity (green outlines) compared to the wild type. FRA-GFP (L, O, green) and E-CAD (M, P, red) in dorsal cells shown in sagittal (L and M) and apical-lateral surface views (O, P). (N, Q) Signal co-localization in merged images (arrows) (see also S4 Fig). Scale bar, 10 μm. Metadata for the graph shown in D can be found at Supporting information S1 Metadata. E-CAD, E-Cadherin; FRA, frazzled; GUKH, guk-holder.

Our results show that there is a noticeable decrease in the size of dorsal cells from early to late stage (Fig 1D and 1E). We also observe that in late stage, dorsal cells are on average 34% smaller than ventral cells (Fig 4A–4D). Since the shape of these cells often approximates a regular hexagon (Figs 1D and 4A), a single dorsal row that shrinks 34% of its area could dislodge 20% of the diameter of a ventral cell or 24% of a dorsal cell. Thus, the constriction of 5 cell rows can roughly pull 1 cell diameter of ventral-sized cells. To test whether the expression of *fra* and *gukh* in dorsal cells are required to constrict and pull lateral cells dorsally, we analyzed if mutants for these genes changed the area of dorsal cells. These experiments reveal that dorsal cells of *fra* mutants are 26% larger than wild-type dorsal cells, whereas *gukh* dorsal cells are almost 40% larger than the wild type (Fig 4D). Thus, we conclude both *fra* and *gukh* are required for the constriction of dorsal cells.

### FRA increases the levels of E-CAD in adherens junctions and GUKH increases F-Actin bundles

While quantifying the cellular areas of *fra* and *gukh* mutants, we noticed that E-CAD at spot adherens junctions (SAJs) appeared much weaker, ill-defined, and with gaps in *fra* mutants, indicating that *fra* is required for maintaining correct E-CAD levels and the integrity of the SAJs (Fig 4F–4G). In contrast, E-CAD levels at the membrane do not appear to change in *gukh* mutants, though there is more diffuse signal within the cells compared to the wild type, as well as abnormal SAJs with fewer interruptions than those seen in *fra* mutants (Fig 4H). In segmented images of E-CAD in the mutants, we note the presence of cells with jagged membranes and fused cells, as opposed to the smooth contours of well-separated cells in the wild type. These differences can be quantified by measuring the circularity of cell contours in *fra* and *gukh* mutants and wild-type embryos (red and green outlines in Fig 4I–4K). The SAJs are apical constrictions containing E-CAD with catenin among other proteins that interact with actin and regulate cell adhesion and signaling [41–43]. Our analyses also revealed that like FRA, E-CAD is more abundant dorsally than ventrally and its expression is greatly reduced in the ventral presumptive mesoderm (Fig 4E and 4F) [44]. This asymmetry of E-CAD is regulated directly or indirectly by DL since dorsalized embryos that lack nuclear DL have high E-CAD levels, and ventralized embryos with ubiquitous nuclear DL have low E-CAD levels (S3 Fig). Previous work on wound healing also found that E-CAD is either directly or indirectly regulated by DL [45]. However, in the case shown here, at least part of this regulation is indirect and mediated by FRA because in the absence of *fra*, the levels of E-CAD throughout the embryo drop to levels found in the ventral region (Figs 4E, 4G and S4). This similar distribution of E-CAD and FRA is consistent with the fact that E-CAD was also identified in our genomic screening for targets of DPP and/or DL. In agreement with these results, we show that both FRA and E-CAD co-localize at SAJs (Figs 4L–4Q and S5). In addition, we show that E-CAD regulation is independent from DPP (S6A Fig). Together, these data indicate that FRA is necessary to maintain high levels of E-CAD at the membrane.

Since GUKH is required for apical cell constriction and its protein contains the SCAR-WAVE domain implicated in the nucleation of actin in filaments [31,46,47], we asked if the distribution of filamentous actin (F-Actin) in the cell perimeter was affected in *gukh* mutants. These analyses show that the loss of GUKH reduces the thickness of the actin bundles localized right below the cell membranes (S7 Fig). Consistent with this result, we show that *dpp* mutants, which lack *gukh* expression in the dorsal midline (Fig 3B), also have thinner actin bundles in the dorsal region (S6B Fig).

Thus, from these experiments, we conclude that GUKH constricts cells by increasing F-actin. The ability of GUKH to regulate cell area appears to be highly conserved since proteins of the SCAR/WAVE family regulate cell morphology from plants to humans by promoting actin filament nucleation [46,47]. For example, the *gukh* human ortholog NHS was shown to maintain cells constricted, and its removal leads to a cell spreading phenotype [36].

### Cell movement is required for the proper formation of gene expression domains

**Fig 5:**
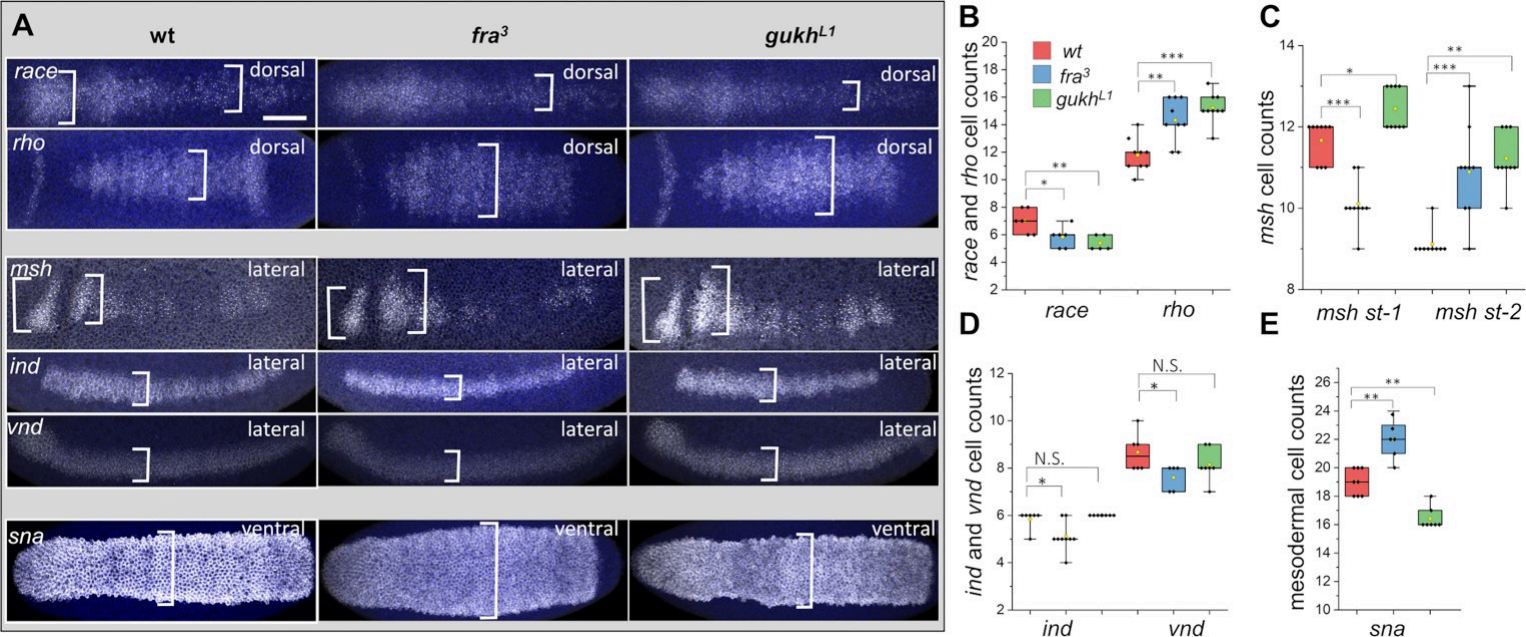
D/V expression domains change in *fra* and *gukh* mutants. (A) RNA in situ for *race*, *rho*, *msh*, *ind*, *vnd*, and *sna* in wild type, *fra*^*3*^ and *gukh*^*L1*^. Brackets, domain width. Cell counts of ectodermal expression genes *race* and *rho* (B), neuroectodermal genes *msh* (C), *ind* and *vnd* (D), and mesodermal gene *sna* (E). Asterisks, threshold values of *p*-values calculated with two-tail Mann–Whitney test (*p < 0.05, **p < 0.01, ***p < 0.0001). N.S., not significant. Yellow dots, mean values. Scale bar, 60 μm. Metadata for the graphs shown in B–E can be found at Supporting information S1 Metadata. D/V, dorso-ventral; FRA, frazzle; GUKH, guk-holder; ind, intermediate nervous system defective; msh, muscle segment homeodomain; sna, snail; vnd, ventral nervous defective.

Thus far, our results reveal how DL and DPP gradients regulate organized cell movements through 2 effector genes that modify cell shape and adhesion, *gukh* and *fra*. However, it is unclear if the cell movement generated by these proteins is required for the proper separation of the embryonic layers in different gene expression domains. To address this issue, we analyzed whether *fra* and *gukh* embryos affect the expression domains of 6 D/V genes involved in ectodermal, neuroectodermal, and mesodermal cell specification. Our results show that the changes in cell density profiles in these mutants affect the specification of all 3 D/V layers (Fig 5A). In the dorsal region, we note that within the nested domains of *race* and *rho*, *race* decreases in size and *rho* is expanded (Fig 5A and 5B). In the neuroectoderm, the *muscle segment homeodomain (msh)* [48] domain is expanded and misshapen in *fra*^*3*^ and *gukh*^*L1*^, whereas the lateral and ventral domains marked by *ind* [49] and *ventral nervous defective (vnd)* [50,51] are reduced in *fra*^3^ (Fig 5A, 5C and 5D). Finally, in the mesoderm, the *sna* domain is expanded in *fra*^*3*^ but reduced in *gukh*^*L1*^ (Fig 5A and 5E). Thus, these results show that the formation of cell density patterns is essential for the correct embryonic patterning.

### The stereotyped cell movements define the precise gradient thresholds required for the separation of gene expression domains

**Fig 6:**
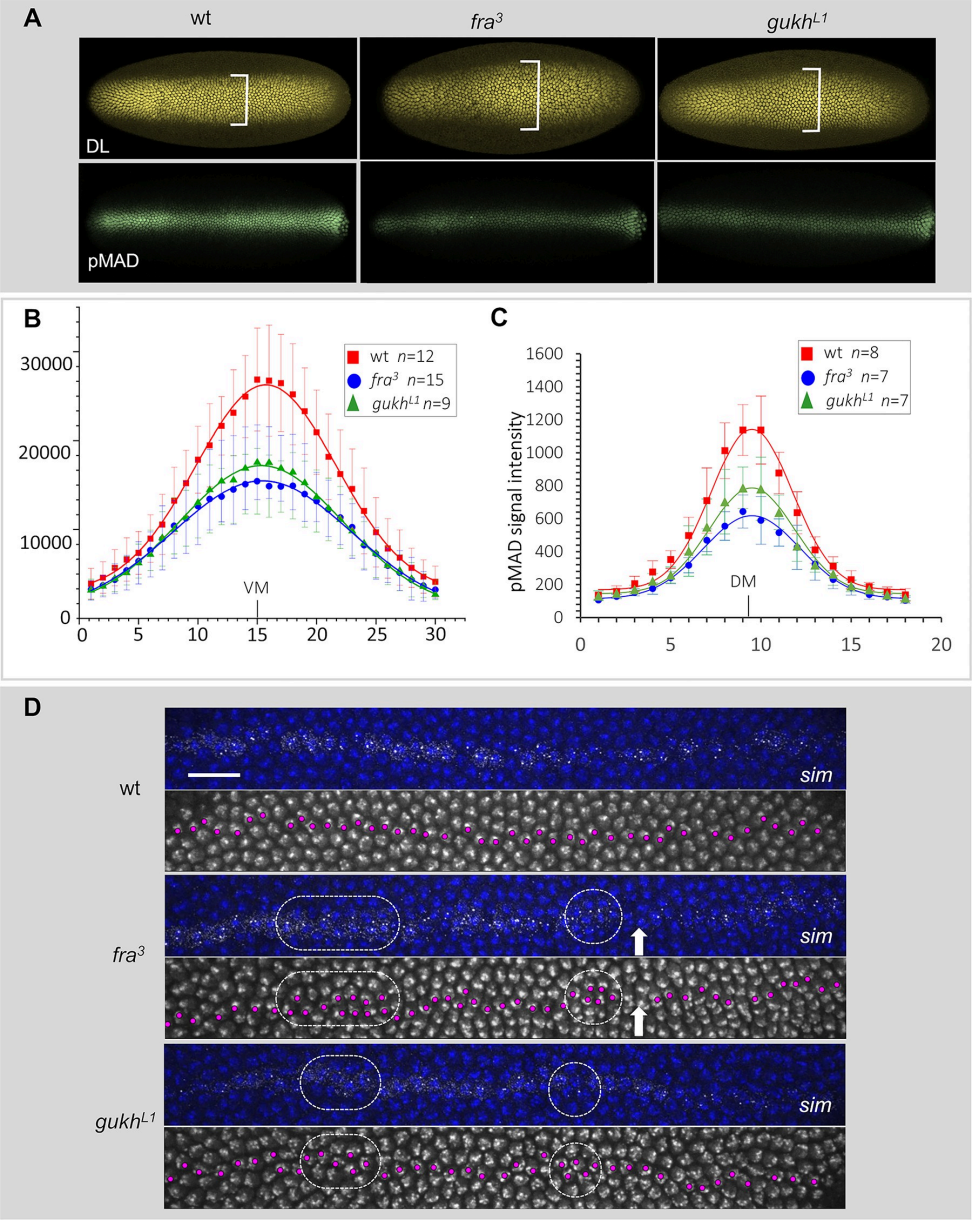
GUKH and FRA modify DL and DPP thresholds levels and displace cells within expression borders. (A–C) Detection and quantification of nuclear DL and pMAD in wild type, *fra* and *gukh*. (A) Ventral and dorsal views of mutant and wild-type embryos stained for anti-DL (yellow) and anti-pMAD (green) antibodies. (B) DL gradient quantification shows lower peak in *fra* (blue) and *gukh* (green) than in wild type (red). VM, ventral midline. (C) pMAD gradient decreases in amplitude in both mutants. DM, dorsal midline. Error bars, standard deviation; n, sample sizes. (D) Mesectodermal border is disrupted in *fra* and *gukh*. *sim* RNA in late-stage embryos stained with Hoescht (gray and blue in alternating panels). Magenta dots, *sim+* nuclei. Wild-type *sim* pattern forms a straight single-cell row. *sim* expression is interrupted in *fra* (arrows) and forms clusters in both *fra* and *gukh* (dashed lines) (see also S7 Fig). Scale bars, 60 μm (A) and 20 μm (D). Metadata for the graphs shown in B and C can be found at Supporting information S1 Metadata. DL, dorsal; DPP, decapentaplegic; FRA, frazzled; GUKH, guk-holder; p-MAD, phosphorylated-Mothers Against DPP.

The finding above implies that the gradients of DPP and/or DL must be affected in *fra* and *gukh* mutants. To directly test this prediction, we first analyzed the distribution of nuclear DL levels in *fra* and *gukh* mutants (Fig 6A). These experiments show that the increase in ventral cell density seen in the heatmaps of both mutants (Fig 3L) involves a broader domain of visible nuclear DL in *fra* and an apparent lower intensity in the midline of both mutants compared to the wild type (Fig 6A). To determine the shape of the DL gradient, we quantified the levels of nuclear DL in these mutants (Fig 6B). These analyses reveal that the DL gradient in *fra* and *gukh* mutants has a pronounced flattened peak in the ventral midline compared to the wild type (Fig 6B). In the regions with lower DL levels (where standard deviations of signal intensities overlap, Fig 6B), we note that DL signal appear fuzzier in *fra* than in the wild type (Fig 6A). This fuzziness suggested that the gradient decays more gradually in *fra*, which could explain the expansion of the *sna* domain, and the normalized gradients confirm this expectation (S8 Fig). Since SNA is a negative regulator of neuroectodermal genes, including *vnd*, the invasion of SNA into the neuroectoderm explains why part of the *vnd* expression in the neuroectoderm is eliminated in *fra* (Fig 5A and 5D) [16,52–56]. In contrast, *gukh* mutants do not have an expansion in SNA and *vnd* and *ind* are not affected (Fig 5A and 5D). Thus, from these experiments, we conclude that the movements of cells exiting from the ventral region in the wild type affects how the DL gradient is laid out.

These experiments suggest that lateral cells of *fra* and *gukh* mutants are more dorsalized due to the reduction in DL levels, which could explain the ectodermal expansion of *rho* into more lateral regions (Fig 5A). However, if this were the case, the reduction of nuclear DL in the mutants should increase the exposure of *msh* to the repressive activity of DPP and cause a retraction of *msh* domain [57,58]. Nevertheless, what we see in both mutants is exactly the opposite, which is a significant expansion of *msh* domain dorsally (Fig 5A and 5C). This result unmistakably shows that in these 2 mutants, DPP reaches the neuroectoderm below the threshold necessary to repress the dorsal boundary of *msh* [58], and therefore, the DPP gradient must be also affected. To confirm this expectation, we analyzed the expression of phosphorylated-Mothers Against DPP (p-MAD), which accumulates in the nucleus in response to DPP [59–61]. We measured nuclear pMAD levels at late cellularization stage, when the DPP gradient becomes stable and nuclear pMAD levels are high and report peak levels of DPP activation within a narrow stripe of dorsal-most cells [60,62–64]. These experiments show that pMAD levels drop dramatically in *fra* and *gukh* mutants (Fig 6A and 6C), which is consistent with the reduction in the *race* domain of both mutants (Fig 5A and 5B). Thus, in these mutants, DPP does not reach its peak levels dorsally and collapses forming a flattened gradient that spreads more laterally as revealed by the expansion of *rho* (Figs 5A, 6A and 6C), and reaches the neuroectoderm at lower levels than normal as seen by the expansion of *msh*. Together, these results reveal an unexpected relationship between cell movement and the formation DPP and DL gradients. Namely, that the thresholds of gene activation or repression elicited by the DPP and DL gradients are tightly coordinated with the movement of cells. In other words, the correct gradient thresholds are only achieved when the cells are moving in defined trajectories in response to these gradients.

### FRA and GUKH are required for defining the separation of juxtaposed expression domains

The experiments above show that GUKH and FRA regulate the fate and position of cells by changing the way gradients and thresholds are spread in space. However, it is unclear if in addition, the movement of these cells is required to fine tune borders of gene expression. To address this issue, we analyzed a particular group of cells bordering the mesoderm and neuroectoderm. At this position, *single minded* (*sim*) is expressed in a single row of mesectodermal cells forming a straight line [65], which depends on the sharp *sna* boundary for precise localization of Notch signaling activation [66–69]. If the formation of a straight line of *sim*-expressing cells depends on a highly coordinated exiting of cells from the mesoderm, then halting the exiting of cells should disorganize the line of *sim*-expressing cells. The analysis of the mesodermal boundary in *fra* and *gukh* mutants shows that the *sna* border becomes jagged (S9 Fig), whereas the *sim* expression has gaps and abnormal clusters containing 2 rows of *sim-*expressing cells (Fig 6D). We conclude that FRA and GUKH are required to maintain sharp boundaries and prevent the intrusion of neighboring cell fates from different expression domains. In sum, our data support a model whereby morphogens control organized cell movements, which are essential for the correct placement of cell fates and for maintaining shapes of the gradients themselves.

## Discussion

**Fig 7:**
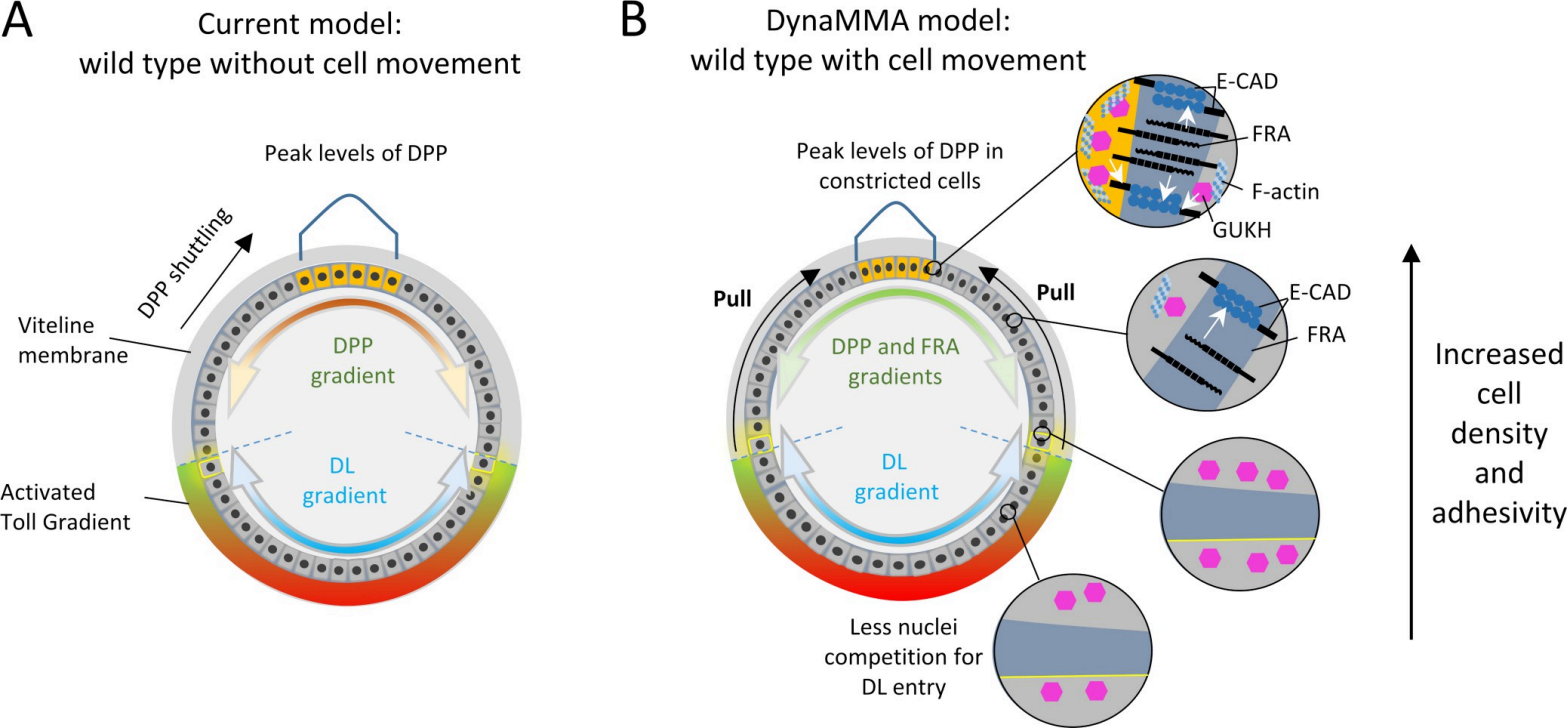
Model of DynaMMA. (A) Current model of embryonic patterning. Cross-section of an embryo in which the cells are static and DPP and DL instruct cells about fate. The gradient of activated Toll receptors regulates the transport of DL to the nucleus and creates a ventral to dorsal gradient of DL. DL represses DPP and restricts its expression to the dorsal side of the embryo. From this position, DPP forms a dorsal-to-ventral gradient that instructs cells. Extracellular shuttling of DPP increases its concentration in the dorsal midline. Note that both gradients are inflexible and cannot tolerate changes in cell position. (B) DynaMMA. In this model, DL excludes DPP and FRA from the ventral domain and creates a dorsal-to-ventral gradient of FRA superimposed to a DPP gradient. High levels of FRA dorsally counteract the cell dissociative and invasive properties that peak DPP levels cause and maintain the cells tightly attached. The gradient of FRA establishes a gradient of cell cohesivity that progressively loosens cells towards more lateral regions. Peak levels of DPP dorsally activate GUKH that constricts the cells dorsally by interacting with F-actin and allows cells to be pulled dorsally. In the DL domain, this pulling allows cells to move up along the ventral–dorsal axis and sharpen the DL gradient through less nuclei competitivity. Similarly, the formation of the DPP gradient also depends on cell densities; in this case, the compression of cells dorsally sharpens the DPP gradient by creating the peak level necessary for patterning dorsal regions of the embryo, possibly through the concentration of TKV receptors in more constricted cells. DL, dorsal; DPP, decapentaplegic; DynaMMA, Dynamic Adjustment of Movement with Morphogenetic Activity; FRA, frazzled; GUKH, guk-holder; TKV, thick veins.

It has been well established that morphogens instruct cells about their fates within tissues by activating and repressing genes at different threshold levels and eliciting local cross-regulation among target genes that help separate domains of gene expression. Extensive evidence shows that morphogens can act over relatively long distances, yet cells can be separated into different genetic programs even when located within few cell diameters apart. If fate separation within neighboring cells per se is remarkable, even more surprising is that morphogens achieve this level of precision while cells are in motion. Indeed, it is difficult to understand how a system of coordinates that seems utterly dependent on protein dispersion can instruct cells in motion with such high level of precision. Our results support a new model that integrates gradient activity and cell position, which we refer to as model of Dynamic Adjustment of Movement with Morphogenetic Activity (DynaMMA, Fig 7) and discuss below.

Current models of DV patterning draw from the considerable effort put forth to explain fate specification and position through mathematical models that test different scenarios involving variables such as dispersion of ligands, production, and degradation rates of pathway components and time (reviewed in [70]). Since these models usually assume that cell fields are static, the variations in the shape of a gradient and gene responses are frequently interpreted in the realm of the dynamics of the components of a pathway, not cells and their spatial positions over time (Fig 7A). The gradient of DL, for example, is generated by the graded activation of Tl receptor in response to a cascade that activates its ligand Spätzle in the perivitelline space. Tl activation causes the degradation of cactus allowing free DL to translocate into the nucleus (reviewed in [71]). In contrast, our results reveal an additional mechanism that is key to the formation of this gradient which is the exiting of cells from the ventral region and their passing through progressively lower levels of active Tl. The cells that remain in the ventral-most region of the embryo are expected to experience an increase in nuclear DL levels due to a decreased competition among nuclei that receive DL. Indeed, it has been shown the DL gradient is sensitive to nuclear density and quickly increases in amplitude during early cellularization stage [72–75]. *fra* and *gukh* mutants clearly lack this increase in amplitude, which can be explained by the increased competition for DL nuclear transport caused by the more densely packed nuclei in the ventral region of these mutants compared to the wild type. The change in nuclear DL gradient caused by the exit of ventral cells towards the dorsal region appears to be an important mechanism that ties DL and DPP to a single coordinate system similar to a tug of war where DPP is on one end pulling cells and DL on the other resisting to this pulling force. This system allows for assigning cells with nuclear DL to specific positions in response to DPP levels, and in these positions, cells acquire fates that depend on both DL and DPP. Since DPP can modulate the exiting of ventral cells from the mesoderm and how much these cells move in and away from the zone of Tl activation, DPP ultimately can regulate the levels of nuclear DL, not transcriptionally, but by cell attraction. This exquisitely simple mechanism of cell–cell interaction is potentially capable of creating very precise variations in target gene expression that are based on the thresholds of 2 morphogens in a single position. With this view in mind, it is inevitable to conclude that cell movement does not pose a hindrance to fate specification, but indeed is the very way of achieving maximal precision in determining the position of cells and their fate (Fig 7B).

### Peak levels of DPP are regulated by cell density

Whereas ventrally the DL gradient can be readjusted by the exit of ventral cells to more lateral regions, dorsally, the shape of the DPP gradient appears to be regulated by the attraction of cells by DPP to the site where its signaling peaks. This is best illustrated by the collapse of peak levels of DPP and the narrowing of the pMAD stripe in *gukh* and *fra*. Since FRA and GUKH are part of a complex of proteins involved in cell adhesion, it seems clear that the normal sudden sharpening of the pMAD stripe depends on these cell contacts (Fig 7B). In particular, the adherens junctions, which have high levels of E-CAD and make strong contacts in the presence of high levels of FRA. Consistent with these observations, FRA co-localizes with E-CAD and the loss of FRA alone eliminates the asymmetric localization of E-CAD along the D/V axis (Figs 4G and S3). Thus, the dorsal-to-ventral gradient of FRA elicits a gradient of E-CAD and these gradients appear to be essential in determining the range of peak DPP levels. The mechanism by which FRA increases E-CAD levels is unknown and needs to be investigated further, but it seems likely that FRA normally might prevent a default degradation of E-CAD. There are at least 3 reasons to believe that the presence of FRA might regulate the degradation of E-CAD. First, it has been shown that cells exposed to high levels of DPP/BMP usually have low levels of E-CAD and high mobility [76,77], which is exactly the opposite of what we have shown in embryos where dorsal cells have high levels of DPP, E-CAD, and FRA. Second, the high levels of E-CAD where DPP peaks can be abolished by removing the FRA receptor alone (Fig 4G). Third, mutants without FRA have low E-CAD levels throughout the embryo comparable to ventral regions without FRA (S3 Fig). Consistent with these observations, it has been reported that human E-CAD is cleaved by Presenilin-1 (PS1), which serves the purpose of disassembling AJs [78]. This is achieved by the binding of PS1 to the GGG binding site in human E-CAD and the cleaving of E-CAD at a γ secretase-like site right at the transmembrane domain on the cytoplasmic side. In *Drosophila*, we note that E-CAD and FRA have the PS1 binding site (1376**GGG**1378 in E-CAD and 1328**GGG**1330 in FRA) and the γ secretase-like site. Thus, FRA appears to have the necessary sequences to compete with E-CAD for the binding to PS1 and in this way reduce E-CAD degradation. Since FRA is regulated by DL and not DPP, FRA becomes expressed where the high levels of DPP would normally lead to a decrease in E-CAD. Such mechanism allows for keeping high levels of DPP signaling without the adverse effects of disassembling cell contacts.

Previous studies have found an association between DPP signaling and E-CAD and suggest that this type of signaling might be recurrently used during development. For example, in the stem cell niche of the *Drosophila* testis, the DPP receptor TKV appears to be guided to the apical region of cells by E-CAD and in this way the signaling is concentrated in a particular region [79]. Similarly, during retinal development, TKV is required for the integrity of cell junctions containing E-CAD and the specification of pigment cells [80]. In addition, the overexpression of E-CAD causes up-regulation of the Punt ortholog TFG-β Receptor II and increases TGF-β signaling in vitro. Finally, E-CAD binds directly to this type II receptor in a complex that includes the type I receptor [81]. Together, these data suggest that FRA might be required to increase the levels of E-CAD and cell tightening, which in turn guides TKV to achieve high levels of DPP signaling. In this view, the lower levels of pMAD in *gukh* and *fra* mutants may be explained by the decrease in cell clustering observed in these mutants (Figs 6C and 7). The role of asymmetric DV cell densities for the formation of peak levels of DL and DPP gradients can be summarized as follows. In the ventral embryonic region, a low cell density is essential to decrease the competition of DL to enter the nuclei, whereas in the dorsal region, a high cell density achieved via cell constriction with tight F-actin bundles and E-CAD membrane localization may contribute to a higher concentration of TKV receptors and DPP peak levels (Fig 7B). Consistent with the possibility that E-CAD is required to generate the peak levels of DPP required to pull cells and create a normal DL gradient, we note that the loss of *e-cad* causes the expansion of *rho* and retraction of *vnd* and *sna* (S10 Fig) that are defects reminiscent of a lower-than-normal DL gradient.

### Dynamic cell interactions are essential for a self-correcting system that organizes tissues and adjusts supply with demand

Polarized gradients in which 1 morphogen antagonizes the other are frequently deployed to establish a series of cell fates within a field of moving cells. Movement is not the exception, but the rule. These facts suggest that after 50 years of the French Flag Model or the French Flag Problem as originally stated [1,4], there is enough evidence that the field of cells that is instructed to differentiate participates in the way this instruction is delivered. Lewis Wolpert, the proponent of the French Flag Model concedes that the current view of morphogen needs additional alternatives that include cell interactions [2]. In a recent work, it was shown that the formation of neural cell fates in the vertebrate neural tube depends on a differential distribution of cadherins established by the Sonic Hedgehog gradient, providing patterning robustness despite cell movements and noisy morphogen signals [82]. Here, we show that cells change their positions in a coordinated fashion in response to morphogenetic gradients. Additionally, cells subjected to the instructive activity of one morphogen can be allocated to different positions by a second morphogen, and in this way, create a continuum of cell fates. Finally, we show that the integrity of this system regulates the shapes of the gradients and therefore how much of the morphogen is delivered. Thus, the separation of different embryonic tissues requires a system that coordinates the levels of morphogen with the position of cells and gene expression.

## Methods

### Fly stocks and genetic crosses

Stocks were obtained from the Bloomington Stock Center (BSC). Wild-type embryos were collected from *y w* flies. *dpp*- embryos were collected from *dpp*^*H46*^ *wg*^*Sp-1*^ *cn*^*1*^ *bw*^*1*^*/*CyO23 stock. *dpp-*, *st2-dpp* embryos were collected from *y w; dpp*^*H46*^ *wg*^*Sp-1*^ *st2-dpp/*CyO stock. *gd*^*7*^ embryos were collected from eggs laid by *v*^*1*^ *gd*^*7*^*/ v*^*1*^ *gd*^*7*^ females. To obtain *gd*^*7*^, *dpp*^*H46*^ double mutant embryos, we generated the stock *v*^*1*^*gd*^*7*^*/*FM3*; dpp*^*H46*^*wg*^*Sp-1*^*cn*^*1*^*bw*^*1*^*/*CyO23 and selected females homozygotes for *gd*^7^ to cross with *dpp*^*H46*^*wg*^*Sp-1*^*cn*^*1*^*bw*^*1*^*/*CyO23 males and collected embryos from this cross. To obtain *gd*^*7*^*; dpp*^*H46*^, *st2-dpp* embryos, females *v*^*1*^ *gd*^*7*^*/ v*^*1*^ *gd*^*7*^*; dpp*^*H46*^ *wg*^*Sp-1*^ *cn*^*1*^ *bw*^*1*^*/*CyO23 were mated to *y w; dpp*^*H46*^ *wg*^*Sp-1*^ *st2-dpp/*CyO males. Embryos with modest nuclear DL concentration were collected from eggs laid by females homozygotes for *Tl*^*r4*^. Embryos with high nuclear DL concentration were collected from eggs laid by females heterozygotes for *Tl*^*10B*^. *fra* mutant embryos were obtained from *fra*^*3*^/CyO, *hb-lacZ* stock. *gukh* mutant embryos were obtained from *gukh*^*L1*^*/*TM3, *hb-lacZ* stock. *gukh*^L1^ was generated by imprecise P-element excision of *gukh*^BG02660^ (w^1118^; P{GT1} *gukh*^BG02660^ line (BSC). *gukh*^L1^ is embryonic lethal and null for *gukh* RNA. Lethality was also confirmed using deficiencies *Df(3R)Exel6182* [FBab0038237] and *Df(3R)BSC474* [FBab0045340]. Embryos expressing GFP-tagged proteins (E-cadherin-GFP and FRA-GFP) were collected from y[1] w*; TI{TI}shg^*GFP*^ [23] and *y[1] w[67c23]; Mi{PT-GFSTF.1}fra[MI06684-GFSTF.1]* [83], obtained from BSC. Fly stocks were maintained at room temperature on a cornmeal-based medium (cornmeal, molasses, yeast, agar, tegosept, and water). Crosses were done at 25°C and 70% humidity.

### Embryo collection and fluorescent multiplex in situ hybridization

Approximately 5 to 6 h embryos were collected from agar plates with grape juice supplemented with yeast at 25°C and fixed according to [84]. Protocols used for fluorescent multiplex in situ hybridization, double in situ and antibody protocols and probe labeling are described in detail in [84]. RNA antisense probes were labeled with Digoxigenin (DIG), Biotin (BIO), Fluorescein (FITC), or Dinitrophenol (DNP) and used at 1:100 concentration. Probes were detected with the following primary antibodies (Sigma-Aldrich): sheep-anti-DIG (1:1,000), goat-anti-BIO (1:1,000), rabbit anti-DNP (1:2,000), and mouse-anti-FITC (1:1,000). Alexa-conjugated secondary antibodies (Invitrogen) were used at 1:500 (Alexa 488, Alexa 555, and Alexa 647). Nuclear staining was done with DAPI or Hoescht. Embryos were mounted in SlowFade Gold mountant (Invitrogen) at −20°C before imaging.

### Antibody immunostaining

The following primary antibodies and concentrations were used: rabbit-anti-Frazzled (1:200, a gift from Dr. Jan lab at University of California San Francisco [25]), rat anti-E-cadherin DCAD2 (1:100, Developmental Studies Hybridoma Bank), mouse anti-β-gal antibody 40-1a (1:1,000, Developmental Studies Hybridoma Bank), chicken anti-β-gal antibody (1:500), Mouse anti-Dorsal 7A4 (1:1,000, Developmental Studies Hybridoma Bank), and rabbit anti-Phospho-Smad1/5 41-D10 (1:800, Cell Signaling Technology, Product #9516T). After incubation, embryos were washed 4 times in PBT for 15 min each and incubated with secondary antibodies diluted at 1:500 for 1 to 1.5 h at room temperature. For F-actin labeling, Phalloidin conjugated with Rhodamin was used at 1:100 in embryos fixed without methanol (Cytoskeleton). Actin was also detected with mouse anti-Actin antibody JLA20 at 1:30 concentration (supernatant form) from the Developmental Studies Hybridoma Bank.

### Confocal microscopy

Embryos were mounted in SlowFade Gold Antifade with added glass beads (Polyscience, 150 to 210 microns, Cat. #05483) to prevent flattening and enable rolling of embryos into desired position. Images were acquired with Zeiss LSM700 confocal microscope Z-stacks using a Plan-Apochromat 20×/0.3 M27, EC Neofluar 40×/1.3 Oil M27 or Plan-Apochromat 63×/1.40 oil M27 objective lenses. Laser power and gain were adjusted within the dynamic range with no signal saturation. For nuclei segmentation of entire embryo surfaces and signal intensity quantifications, Z-stacks of about 30 slides for the embryo dorsal surface and 40 slices for ventral surface were obtained at 1.5 μm Z-step, line average 2, 1.58 μs speed, 1,024 × 1,024 pixels, 12 bits, and 20× objective lens. D/V orientation of embryos was defined by the gene expression patterns of *rho*, *sna*, and *ind*; A/P markers used were *ftz* and *eve* [49,54,85–87]. Ventralized (*Tl* mutants) and dorsalized embryos (*gd* mutants) do not show a patterned gene expression along the D/V axis. However, the geometry of embryo (shorter dorsal side and longer ventral side) and location of pole cells at a more dorsal region are preserved and were used for orienting the embryos. Imaging for segmentation of cell surfaces and measuring of cell size and E-CAD signal intensity in *fra* and wild-type embryos was done with 40× oil objective lens at 0.8 μm Z-step with settings above. For measurements of intensity levels of pMAD and DL, images were collected with 12 bits (pMAD) and 16 bits (DL). Line average 4 and 3.16 μs speed were used for some figure panels to improve visualization.

### Time-lapse imaging

Embryos expressing *shg/e-cad*-*GFP* constructs were collected at 3 to 4 h after being laid on a grape plate and transferred to a double-sided tape and dechorionated by hand using a needle. The dechorionated embryo was placed on a coverslip covered with Heptane glue and inverted on a gas permeable Lumox culture dish (Greiner) covered with Halocarbon oil. Time-lapse imaging was initiated right before cellularization for about 1 to 1.5 h of duration. Z-stacks were set up for 3-min intervals. After imaging, embryos were unmounted, transferred to a slide with halocarbon oil, placed inside a humid chamber, and allowed to develop at 25°C. Videos from embryos that hatched to the first instar larva stage were used for analysis.

### Image segmentation of nuclei and cell density spatial analyses

Z-stacks confocal images of nuclei stained with Hoechst or DAPI were segmented using Fiji software version 1.49s. For segmentation of the embryo dorsal surface, the first 20 slices from the confocal Z-stacks were used from embryos of all genotypes. For the ventral side, the first 25 slices for *gd*^*7*^, *gd*^*7*^*;dpp-* and *gd*^*7*^*;dpp- st2-dpp* embryos and the first 35 slices for embryos of all other genotypes were used. Accuracy of segmentation confirmed with manual nuclei counts was between 90% and 100%. The last few slices of the confocal Z-stacks containing the embryo periphery were discarded, since this region is not segmented reliably [88]. The Z-stack subsets were processed using a “median 3D filter” with x, y, z radius set to 2.0, “gaussian 3D filter” with x, y, z radius set to 1, and “unsharp mask” filter (1.4 radius and 0.7 mask weight for the embryo dorsal surface images; 1.2 and 0.6 for ventral surface images). For dorsal side images of wild-type embryos, an additional “maximum 3D” filter with x, y, z radius 1.0 was applied to improve segmentation in areas with high nuclei densities. All images were filtered 1 last time using sharpen process. The processed images were segmented using the 3D iterative threshold segmentation available in the 3D plugin package [89,90]. The minimum volume was set at 15 μm for ventral and dorsal embryo surfaces and the maximum volume was set at 190 μm for dorsal surface and 200 μm for ventral surface. “Criteria method” was set as “volume,” “threshold method” was set as “kmeans,” “value method” was set at 100, “minimum threshold” was set as default, and “filtering box” was selected. After segmentation, 3D object counter was used to obtain centroids of segmented nuclei and their x, y, z coordinates. In order to identify most cells with least background noise, a threshold value between 40 and 100 was selected in the 3D object counter window depending on the embryo image. To obtain the signal intensity of some of the markers (e.g., *ftz*, *dpp*, and *rho*) within corresponding expressing nuclei objects, we used the option redirect to original confocal image in the 3D object counter settings. Next, the resulting.csv files containing the centroid x, y, z coordinates were imported into Esri ArcMap software to generate the density heatmaps and contour plots. First, a surface mask was created using the “aggregate points” function using an aggregation distance 30. Next, the heatmaps were generated using “kernel density” function and the symbology applied was a “stretched” color-map with high/low values set as 0.0105 and 0.002. The contour plots were set with an interval of 0.0005. For creating average heatmaps of embryos of same genotype, 4 coordinates of points (i.e., anterior and posterior poles, and lateral-most points at the center of the embryo) were matched into the same location across different embryos using “add control points” function in “georeferencing” tool. The average heatmap was then generated using the “raster calculator” under “Map algebra” in “spatial analysis” tools. The symbology applied and contour plot settings used were the same as above.

### Cell counts and statistical analyses

For comparing cell counts at the dorsal and ventral midline regions, Matlab was used to identify the centroid of the projected confocal Z-stack of ventral and dorsal embryo surfaces and to crop a rectangle of 300 × 100 pixels (156.14 × 52.04 μm). The number of nuclei from this rectangle was recorded. The two-tailed Mann–Whitney test was used to test whether dorsal-ventral cell number differences varied among embryos of different genotypes. The two-tailed Wilcoxon signed-rank test was used to test whether cell numbers varied from dorsal to ventral within embryos of the same genotype. For cell counts of *st2-dpp* expressing embryos, regions of interest of 100 × 100 pixels (52.05 × 52.05 μm) were cropped from stripe 2 *ftz* region and in between stripes 6 and 7 along the dorsal midline using Matlab. The cell numbers within these regions of interest were obtained and compared using a two-tailed Wilcoxon signed-rank test. For D/V gene expression domain analyses, two-tailed Mann–Whitney test was used to compare cell counts. In all cases, we used a *p*-value of 0.05 to reject the null hypothesis and accept the alternative hypothesis that the 2 compared data sets are significantly different with a 95% confidence interval.

### Spatial statistics for hot spot analyses

To identify statistically significant cell clusters of high and low density versus nonsignificant randomly distributed cells, Getis-Ordi G* spatial statistic was applied to nuclei centroids of dorsal and ventral embryo surfaces using Esri ArcPro or ArcMap software. The centroids were split using a grid system of size 20 and the “hot spot analysis” tool was used with a fixed distance band of 50 for the denser dorsal embryo surface and 120 for the less dense ventral surface. Grids identified as hot spots are labeled according to their confidence intervals (dark red, 99%; red, 95%, light red, 90%) and the same for cold spots (dark blue, 99%; blue, 95%; light blue, 90%). Nonsignificant grids (NS) with randomly distributed cells are labeled in yellow.

### *fra* and *gukh* identification in genomic in silico screenings

Three main criteria were established to identify our candidate genes. First, a genome-wide search was conducted to select genes with similar expression pattern to the DPP receptor *thickveins* throughout development. This search was done using the mRNAseq data from “modENCODE Temporal Expression Data” [91], deposited in Flybase database (Flybase release FB2014_03May, Dmel 5.57, available in Flybase archives). Second, out of 101 genes identified in this search, we selected those genes with previously described or predicted functions related to cell migration, cell adhesion, or cytoskeleton regulation. Finally, we selected genes with asymmetric expression patterns along the D/V axis according to the expression pattern database maintained by the Berkeley Drosophila Genome Project [92,93]. *gukh* was the first hit on the list of genes with developmental expression pattern similar to *tkv* with a 93.66% of similarity; *fra* was the sixth hit with 89.84% of similarity. Both genes fit the other 2 criteria established.

### *fra* RNA and E-CAD dorso-ventral signal intensity analysis

Individual embryos stained with *fra* antisense probe or anti-E-CAD antibody were imaged on the dorsal, lateral, and ventral sides using the confocal settings described above. The Z-stacks from ventral, dorsal, and lateral surfaces were projected in 2D using maximum intensity projection and the average intensity level and standard deviation from a region of 50 × 50 pixels (26.03 × 26.03 μm) was obtained using Fiji software. Intensity levels of *fra* RNA were measured from 5 D/V locations and were fit to a Gaussian curve using *vnd* as a D/V marker. The measurement of E-CAD intensity was obtained from the centermost region of dorsal, lateral, and ventral projected images and a linear fit was performed. Curve fittings were performed using OriginLab or Excel software.

### Measurement of pMAD and DL gradients

The DL gradient was measured and normalized according to [94]. Briefly, embryos stained with anti-DL antibody and *sog* RNA (used as a D/V marker) were hand-sliced using a 26G 3/8-inch needle at approximately 35% and 65% AP positions to obtain cross-sections of the trunk region [95]. The cross-sections were flipped up and imaged using the same confocal settings for all genotypes (see description of confocal settings above). A small circle of approximately 10 μm^2^ was selected within the 30 ventral-most nuclei and the DL average signal intensity levels were obtained in Fiji. The pMAD gradient was measured in the 18 dorsal-most cells of the embryo. Confocal Z-stacks were projected using maximum intensity 2D projection and a region of interest of 10.4 μm^2^ was cropped inside each of the 18 nuclei and the pMAD signal intensity level was obtained using Fiji software. A Gaussian curve fit for pMAD and DL gradients were obtained using OriginLab or Excel software.

## Supporting information

S1 Video Dorsal surface of a wild-type embryo expressing E-CAD-GFP during cellularization. Note constriction of dorsal-most cells.
(MP4)

S2 Video Time-lapse and segmented surfaces of 7 individual dorsal cells expressing E-CAD-GFP. The first part of this video shows the GFP channel overlaid with the segmented surfaces of seven cells colored in white, which was used to quantify the surface areas over time. The second part of the video shows all cell membranes segmented overlaid with the segmented surfaces of seven cells, each with a different color corresponding to the graph shown in S1 Fig.
(MP4)

S1 Fig Time-lapse imaging of individual dorsal cells expressing E-CAD-GFP shows successive cell constriction over time. Measurements of surface areas of 7 individual dorsal cells that were segmented from S2 Video. Segmentation mask of each individual cell above is shown in the second part of video using the same corresponding colors as seen in the graph. Metadata for the graph shown in this figure can be found at Supporting information S1 Metadata.
(TIF)

S2 Fig Additional information on *gukh* expression pattern and *gukh* and *fra* regulation by *dpp*. Related to Fig 3. (A, B) *gukh* is expressed in the ventral region of the neuroectoderm. Lateral view of a late blastoderm wild-type embryo showing a strong band of *gukh* expression within the ventral neuroectoderm (A) that co-localizes with rhomboid expression (B, rho in magenta, *gukh* in gray). (C, D) Ectopic *dpp* expression restores gukh dorsal expression in *dpp*- embryos (related to Fig 3A and 3B). (C) Dorsal view of a *dpp*-, st2-*dpp* embryo stained for *gukh*. Arrows shows presence of *gukh* nascent transcripts near the source of DPP expression, see high magnification inset. (D) Same embryo showing expression of *gukh* (green), *dpp* (red), and the DPP-target. (E–H) fra is not regulated by DPP. Late blastoderm embryos shown in dorsal view (E, F) and lateral view (G, H). *fra* expression is similar in wild type (E, G) and *dpp*- embryos (F, H).
(TIF)

S3 Fig Cell density heatmaps from lateral region of wild type (wt), *fra*^*3*^, *gukh*^*L1*^embryos; related to Fig 3. Note that cell density is higher in mutants than in the wild type. Green dots indicate position of ventral and dorsal border of the lateral neuroectodermal domain marked by expression of *ind*. *n* indicates number of segmented embryos used for creating the average heatmaps.
(TIF)

S4 Fig E-CAD levels and cellular localization are modified in fra mutants and in embryos without the DL gradient; related to Fig 4. (A) Wild-type levels of E-CAD decrease sharply from dorsal to ventral regions of the embryo (black circles). In *fra*^*3*^ mutants, E-CAD levels decrease in dorsal and lateral regions (squares). In *gd*^*7*^ dorsalized embryos, E-CAD levels are intermediate to high across the entire D/V axis (gray lozenge), whereas in *Tl*^*10b*^ ventralized embryos, E-CAD levels are low across the entire embryo (gray triangle). (B) Measurements of E-CAD intensity levels in small regions from one side of cell across the other side, spanning the membranes and intracellular regions. E-CAD intensity levels in the membrane are lower in the mutants compared to the wild type but more intense within the intracellular regions than in the wild type. This pattern is consistent with a more diffuse staining of E-CAD within the cell and less localized signal at the membrane. Error bars, standard deviation. Sample size *n* = 9. Metadata for the graphs shown in A and B can be found at Supporting information S1 Metadata.
(TIF)

S5 Fig Distribution of FRA protein across apical to basal regions during late cellularization stage. (A, B) Localization of FRA-GFP, DLG, and E-CAD. (A) Staining of FRA-GFP (green) and discs large (DLG, magenta). (B) FRA-GFP (green) and E-CAD (magenta). Top 2 rows show sagittal view of dorsal cells and ventral cells. Note high levels of FRA in the apical region of dorsal cells. Bottom 3 rows show surface view of apical, baso-lateral, and basal regions. Note enrichment of FRA-GFP in cell vertices at baso-lateral region and co-localization with E-CAD (see Fig 4 in main paper). (C) Similar staining patterns are confirmed with anti-FRA antibody (green) and anti-E-CAD antibody (magenta) at the baso-lateral region of dorsal cells. Note co-localization of FRA and E-CAD to the cell vertices (gray in merge image).
(TIF)

S6 Fig *dpp* mutants have normal E-CAD levels and thinner Actin filaments. (A) E-CAD intensity levels in dorsal regions of wild type (yw) and *dpp* mutant embryos are similar. (B) Actin filament thickness is significantly smaller in *dpp* mutants than in the wild type (yw). Embryos were stained with anti-Actin antibody and measurements were taken at approximately 3.5–4 microns from the apical region of dorsal cells; *p*-values calculated with two-tail Mann–Whitney test. Metadata for the graphs shown in A and B can be found at Supporting information S1 Metadata.
(TIF)

S7 Fig GUKH is required for proper formation of F-Actin bundles; related to Fig 4. (A–F) Phalloidin staining in late cellularization stage of wild type (A–C) and *gukh* mutants (D–F). A single confocal focal plane at approximately 6 μm from the apical region is shown for cells from dorsal (A, D), lateral (B, E), and ventral (C, F) regions of the embryo are shown. Note decreased levels and thinner bundles in *gukh* compared to wild type. (G) Quantification of fiber thicknesses in wild type and *gukh* in dorsal, lateral, and ventral regions. Error bars, standard deviation. Asterisks indicate threshold values for statistical tests based on *p*-values calculated with two-tail Mann–Whitney test (***p < 0.0001). Sample size *n* = 60 for individual measurements (dorsal, lateral, and ventral) in wild type and *gukh*. Metadata for the graph shown in G can be found at Supporting information S1 Metadata.
(TIF)

S8 Fig The normalized DL gradient in wild type (red squares) and *fra* mutant (blue circle) show a more gradual decay in DL intensity levels in *fra* compared to the wild type; related to Fig 6. Metadata for this graph can be found at Supporting information S1 Metadata (please refer to data for Fig 6B).
(TIF)

S9 Fig SNA expression border becomes jagged in the absence of FRA or GUKH; related to Fig 6. *sna* RNA in situ staining of late cellularization stage embryos show a straight border in wild-type embryos (A), but irregular border in *fra* (B) and *gukh* (C) embryos (arrows).
(TIF)

S10 Fig *ecad* affects fate specification. Cell counts of *race* and *rho* (ectoderm), *ind* and *vnd* (neuroectoderm), and *sna* (mesoderm), show that *ecad* mutants (blue) have enlarged *rho* domain and reduced *vnd* and *sna* domains compared to the wild type (red); *p*-values indicated on graphs were calculated with two-tail Mann–Whitney test. Metadata for the graph shown in this figure can be found at Supporting information S1 Metadata.
(TIF)

S1 Table DL and MAD binding sites present in *gukh* and *fra*. (XLSX)

S1 Metadata Excel file containing metadata for this paper. (XLSX)

## Abbreviations

3D: three dimensional
A/P: antero-posterior
BMP-4: bone morphogenetic 4
DL: dorsal; DPP, decapentaplegic
D/V: dorso-ventral
DynaMMA: Dynamic Adjustment of Movement with Morphogenetic Activity
E-CAD: E-Cadherin
FRA: frazzled
FTZ: fushi tarazu
GD: gastrulation defective
GIS: geographic information system
GUKH: guk-holder
ind: intermediate nervous system defective
MAGUK: membrane-associated guanylate kinase
msh: muscle segment homeodomain
NHS: Nance–Horan syndrome
p-MAD: phosphorylated-Mothers Against DPP; rho, rhomboid
SAJ: spot adherens junction
sim: single minded; sna, snail
sog: short gastrulation
ST2: even skipped stripe 2
TKV: thick veins
vnd: ventral nervous defective
