# Supplementary figures and images for: The epithelial polarity genes frazzled and GUK-holder adjust morphogen gradients to coordinate changes in cell position with cell fate specification
Source: PLoS Biol. 2023 Mar 13;21(3):e3002021. doi: 10.1371/journal.pbio.3002021 (PMC10035841; doi:10.1371/journal.pbio.3002021)

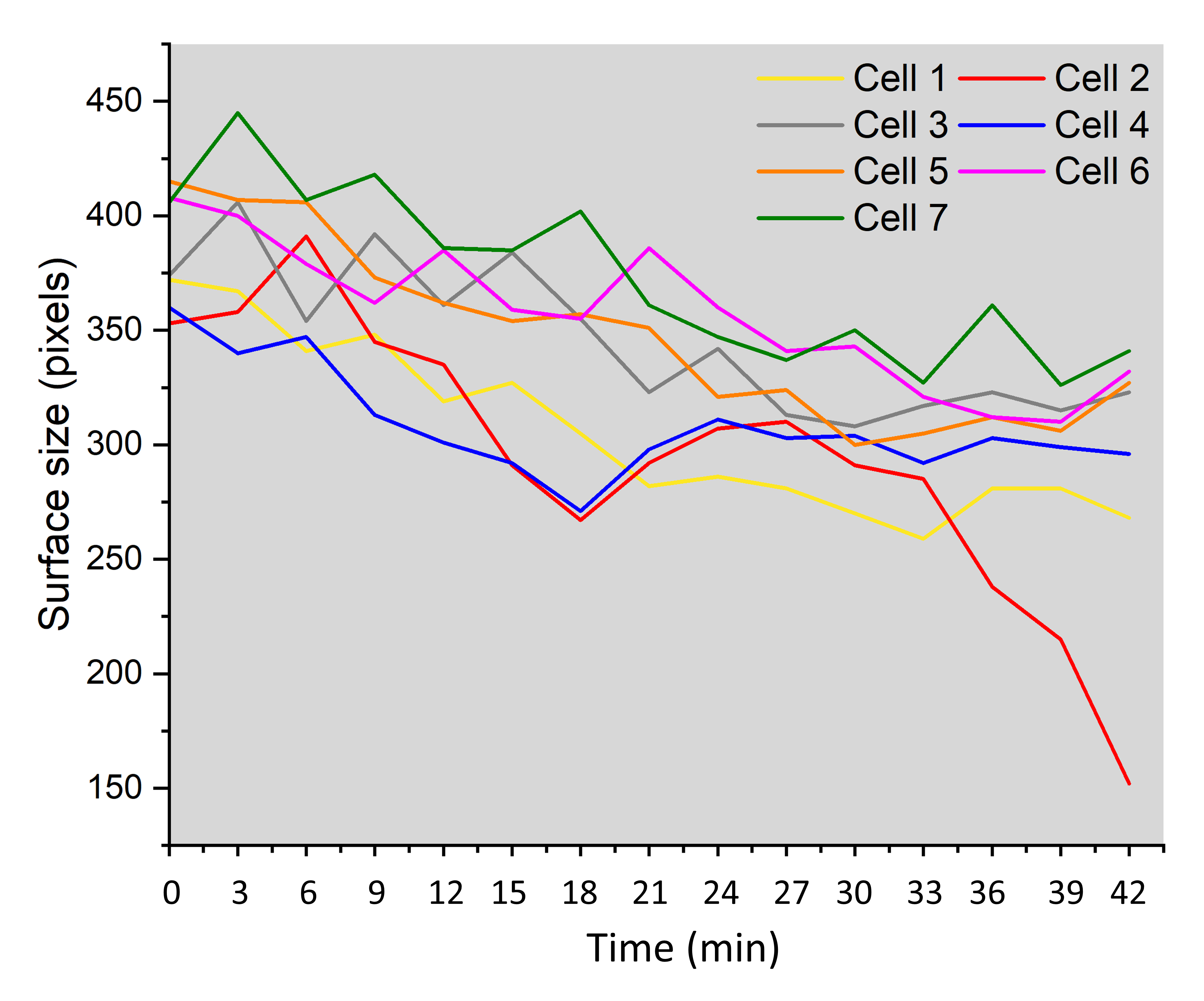

Supplement: S1 Fig — Measurements of surface areas of 7 individual dorsal cells that were segmented from S2 Video. Segmentation mask of each individual cell above is shown in the second part of video using the same corresponding colors as seen in the graph. Metadata for the graph shown in this figure can be found at Supporting information S1 Metadata. (TIF) [file pbio.3002021.s003.TIF]

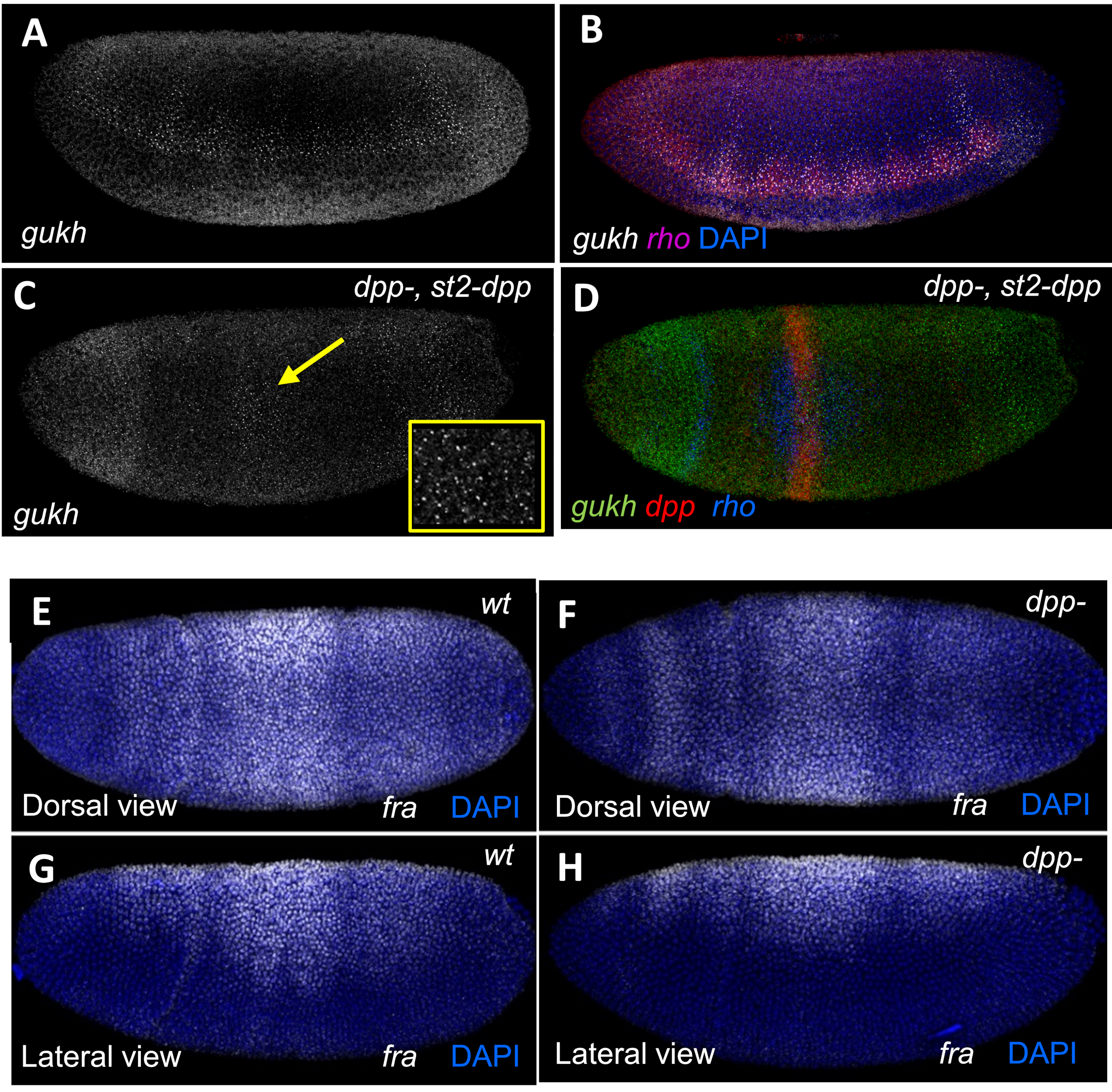

Supplement: S2 Fig — (A, B) gukh is expressed in the ventral region of the neuroectoderm. Lateral view of a late blastoderm wild-type embryo showing a strong band of gukh expression within the ventral neuroectoderm (A) that co-localizes with rhomboid expression (B, rho in magenta, gukh in gray). (C, D) Ectopic dpp expression restores gukh dorsal expression in dpp- embryos (related to Fig 3A and 3B). (C) Dorsal view of a dpp-, st2-dpp embryo stained for gukh. Arrows shows presence of gukh nascent transcripts near the source of DPP expression, see high magnification inset. (D) Same embryo showing expression of gukh (green), dpp (red), and the DPP-target. (E–H) fra is not regulated by DPP. Late blastoderm embryos shown in dorsal view (E, F) and lateral view (G, H). fra expression is similar in wild type (E, G) and dpp- embryos (F, H). (TIF) [file pbio.3002021.s004.TIF]

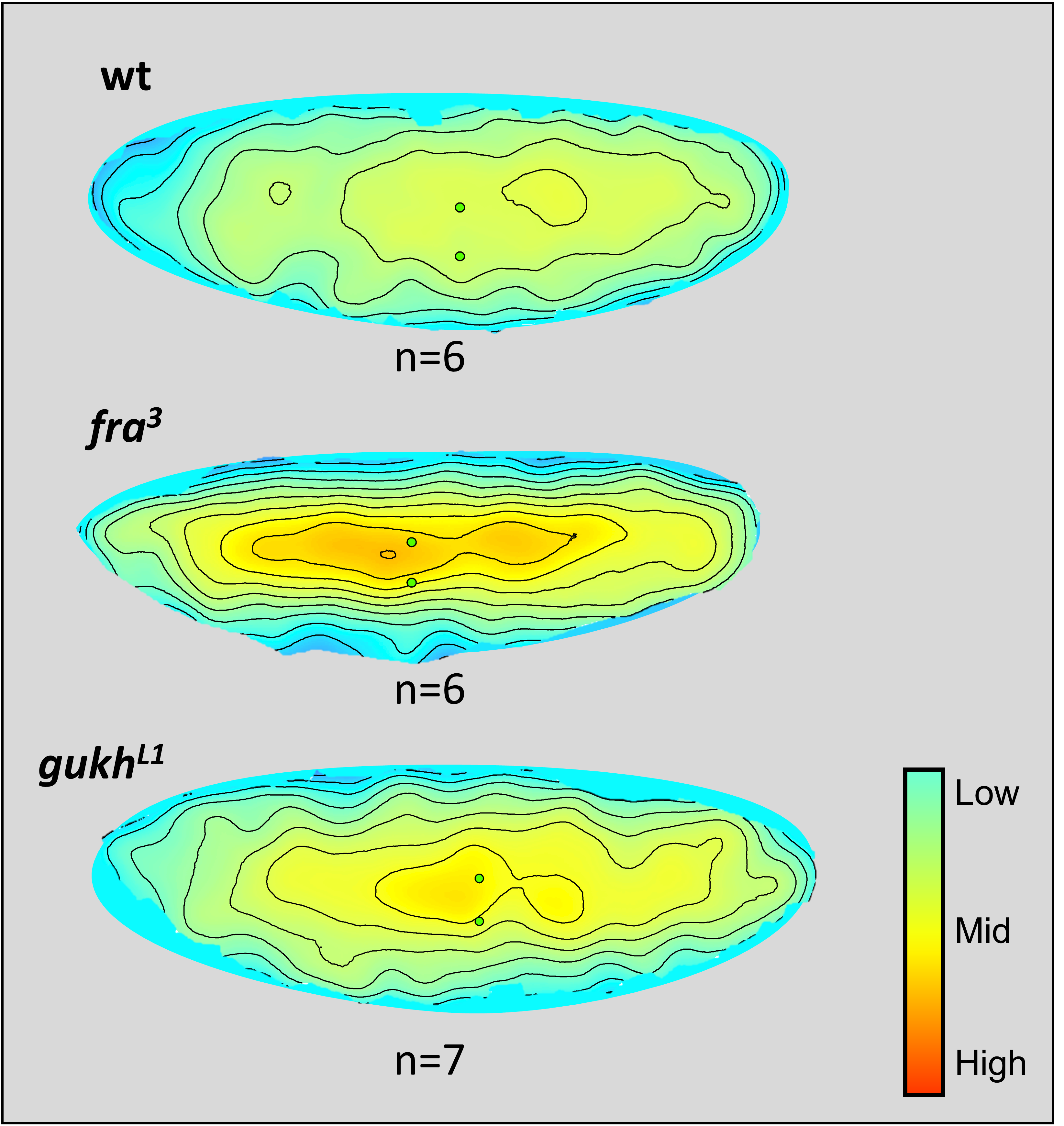

Supplement: S3 Fig — Note that cell density is higher in mutants than in the wild type. Green dots indicate position of ventral and dorsal border of the lateral neuroectodermal domain marked by expression of ind. n indicates number of segmented embryos used for creating the average heatmaps. (TIF) [file pbio.3002021.s005.TIF]

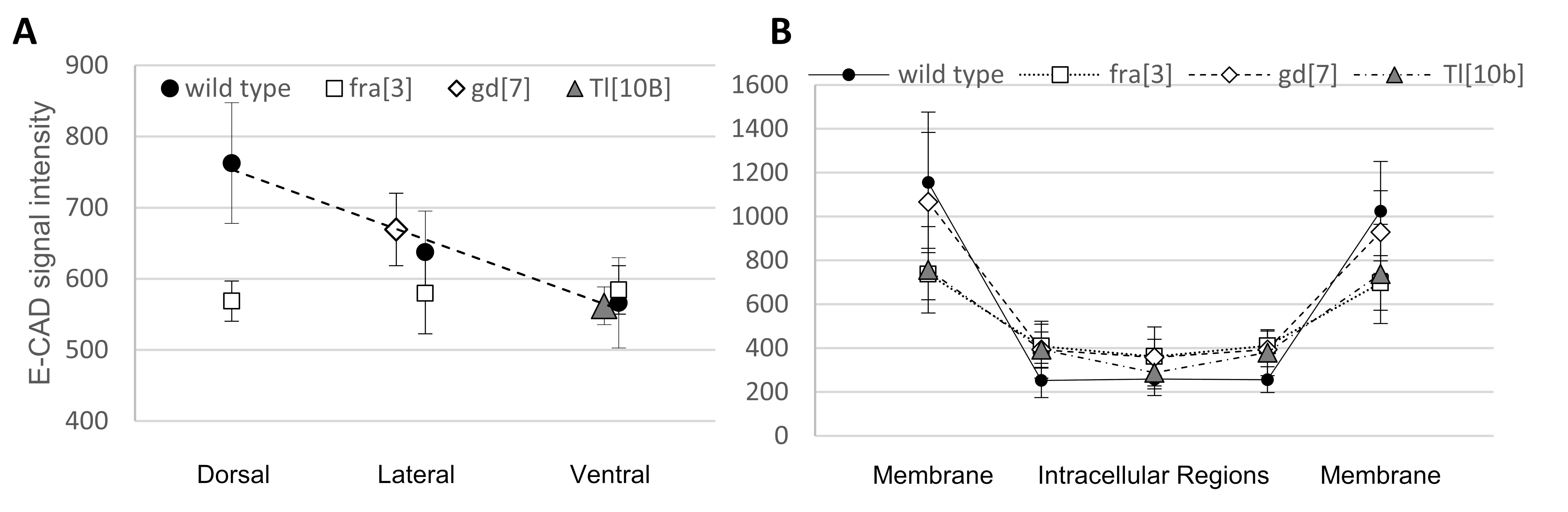

Supplement: S4 Fig — (A) Wild-type levels of E-CAD decrease sharply from dorsal to ventral regions of the embryo (black circles). In fra3 mutants, E-CAD levels decrease in dorsal and lateral regions (squares). In gd7 dorsalized embryos, E-CAD levels are intermediate to high across the entire D/V axis (gray lozenge), whereas in Tl10b ventralized embryos, E-CAD levels are low across the entire embryo (gray triangle). (B) Measurements of E-CAD intensity levels in small regions from one side of cell across the other side, spanning the membranes and intracellular regions. E-CAD intensity levels in the membrane are lower in the mutants compared to the wild type but more intense within the intracellular regions than in the wild type. This pattern is consistent with a more diffuse staining of E-CAD within the cell and less localized signal at the membrane. Error bars, standard deviation. Sample size n = 9. Metadata for the graphs shown in A and B can be found at Supporting information S1 Metadata. (TIF) [file pbio.3002021.s006.TIF]

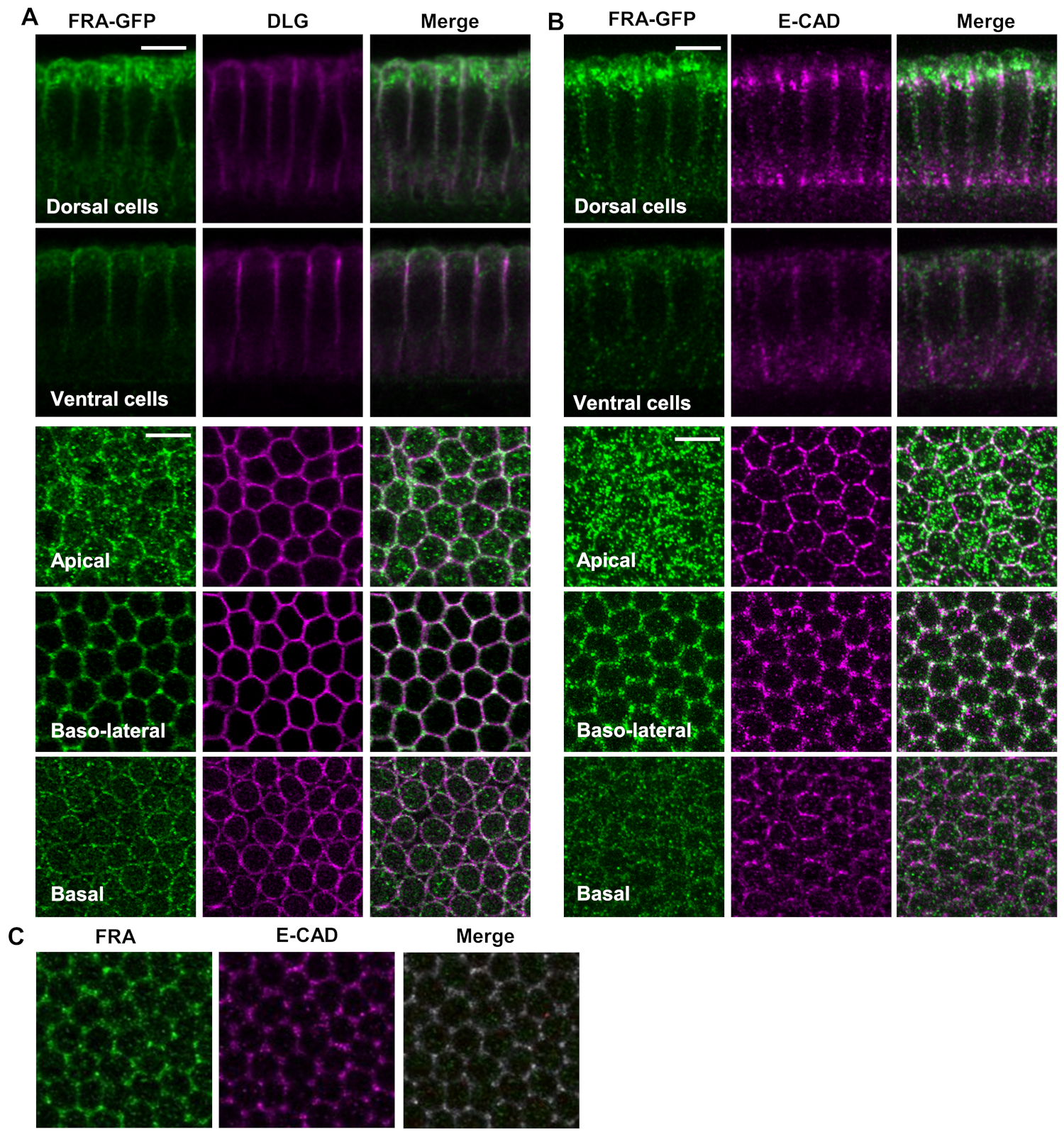

Supplement: S5 Fig — (A, B) Localization of FRA-GFP, DLG, and E-CAD. (A) Staining of FRA-GFP (green) and discs large (DLG, magenta). (B) FRA-GFP (green) and E-CAD (magenta). Top 2 rows show sagittal view of dorsal cells and ventral cells. Note high levels of FRA in the apical region of dorsal cells. Bottom 3 rows show surface view of apical, baso-lateral, and basal regions. Note enrichment of FRA-GFP in cell vertices at baso-lateral region and co-localization with E-CAD (see Fig 4 in main paper). (C) Similar staining patterns are confirmed with anti-FRA antibody (green) and anti-E-CAD antibody (magenta) at the baso-lateral region of dorsal cells. Note co-localization of FRA and E-CAD to the cell vertices (gray in merge image). (TIF) [file pbio.3002021.s007.TIF]

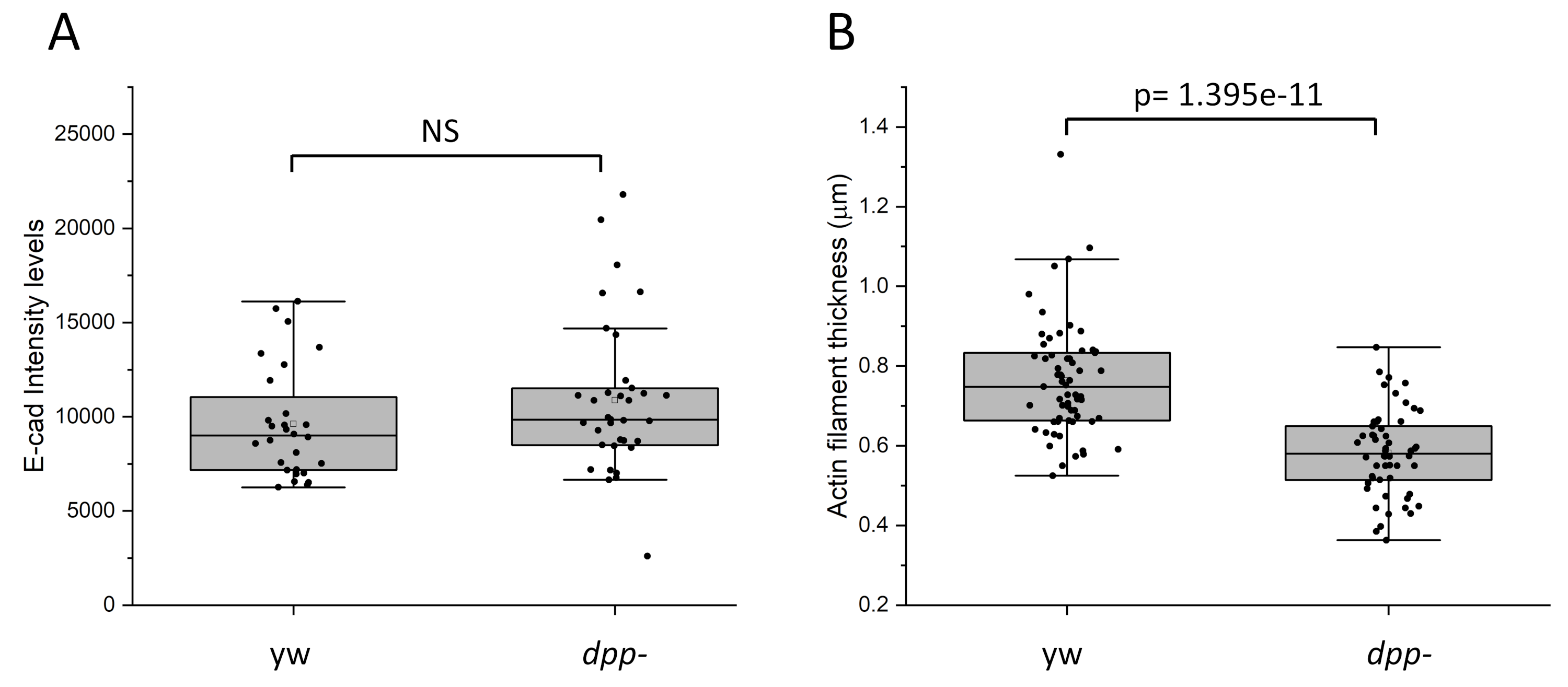

Supplement: S6 Fig — (A) E-CAD intensity levels in dorsal regions of wild type (yw) and dpp mutant embryos are similar. (B) Actin filament thickness is significantly smaller in dpp mutants than in the wild type (yw). Embryos were stained with anti-Actin antibody and measurements were taken at approximately 3.5–4 microns from the apical region of dorsal cells; p-values calculated with two-tail Mann–Whitney test. Metadata for the graphs shown in A and B can be found at Supporting information S1 Metadata. (TIF) [file pbio.3002021.s008.TIF]

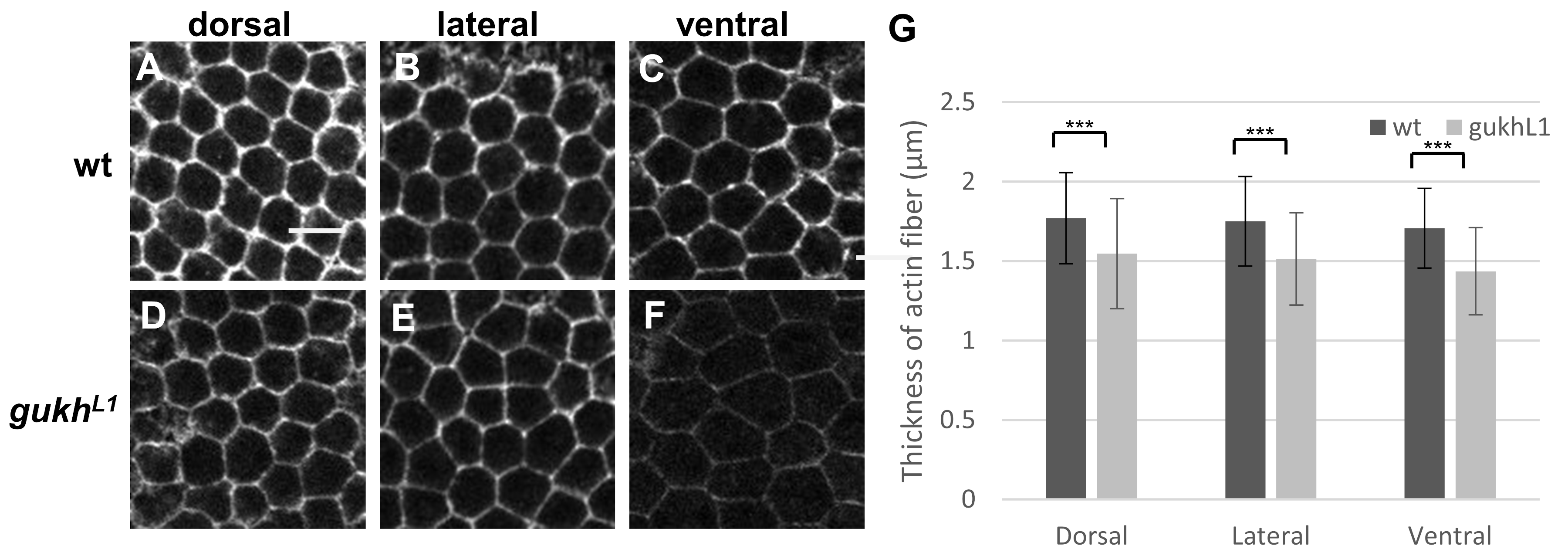

Supplement: S7 Fig — (A–F) Phalloidin staining in late cellularization stage of wild type (A–C) and gukh mutants (D–F). A single confocal focal plane at approximately 6 μm from the apical region is shown for cells from dorsal (A, D), lateral (B, E), and ventral (C, F) regions of the embryo are shown. Note decreased levels and thinner bundles in gukh compared to wild type. (G) Quantification of fiber thicknesses in wild type and gukh in dorsal, lateral, and ventral regions. Error bars, standard deviation. Asterisks indicate threshold values for statistical tests based on p-values calculated with two-tail Mann–Whitney test (***p < 0.0001). Sample size n = 60 for individual measurements (dorsal, lateral, and ventral) in wild type and gukh. Metadata for the graph shown in G can be found at Supporting information S1 Metadata. (TIF) [file pbio.3002021.s009.TIF]

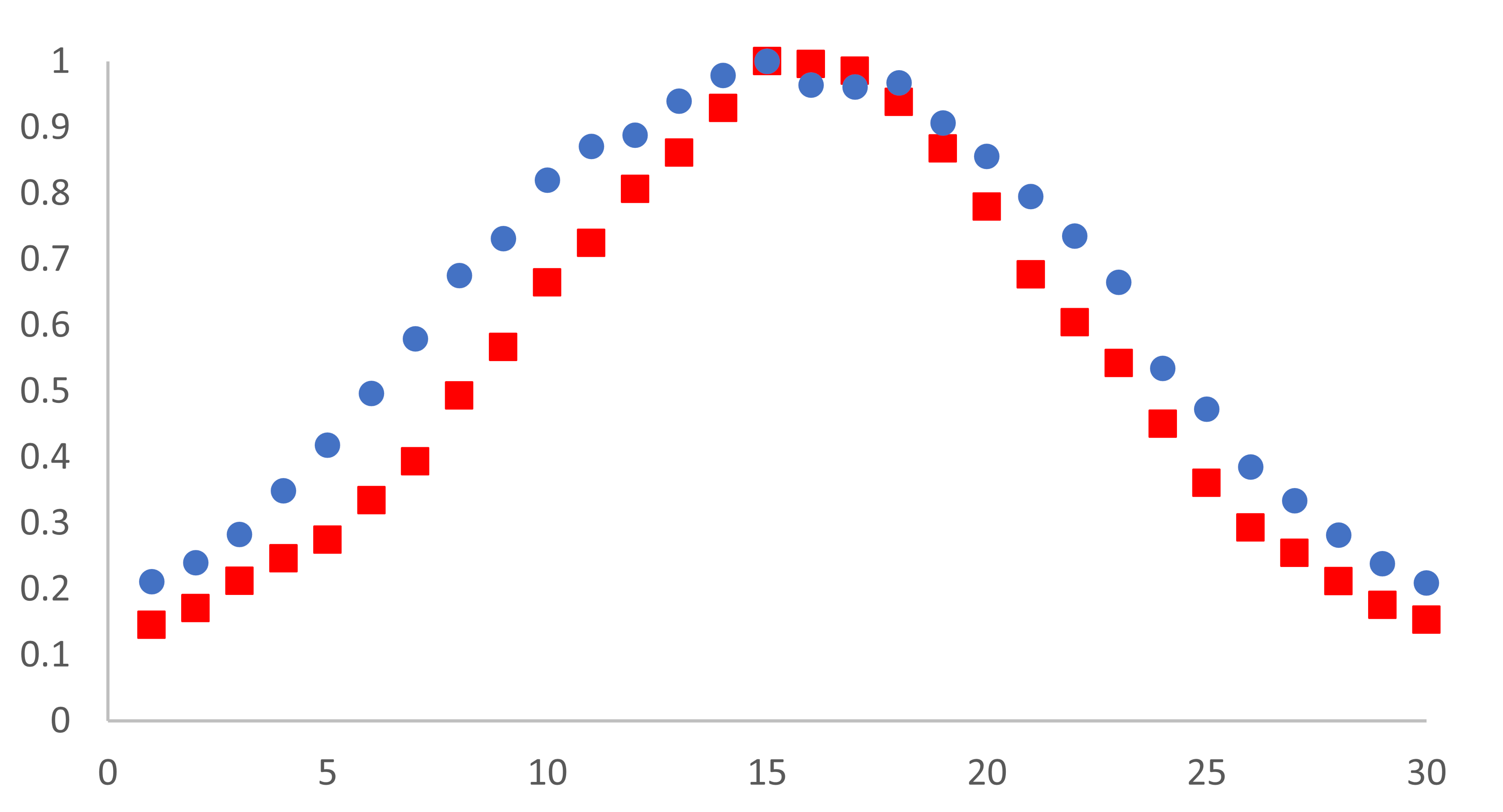

Supplement: S8 Fig — Metadata for this graph can be found at Supporting information S1 Metadata (please refer to data for Fig 6B). (TIF) [file pbio.3002021.s010.TIF]

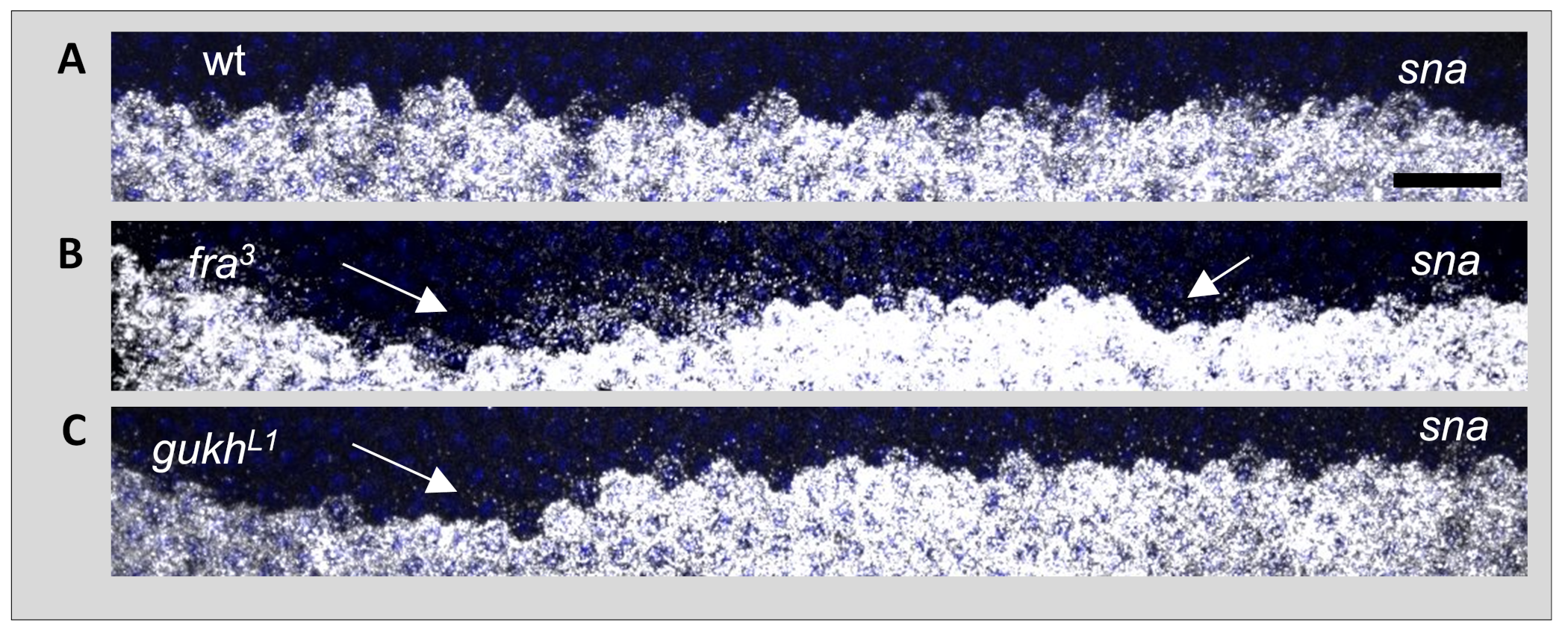

Supplement: S9 Fig — sna RNA in situ staining of late cellularization stage embryos show a straight border in wild-type embryos (A), but irregular border in fra (B) and gukh (C) embryos (arrows). (TIF) [file pbio.3002021.s011.TIF]

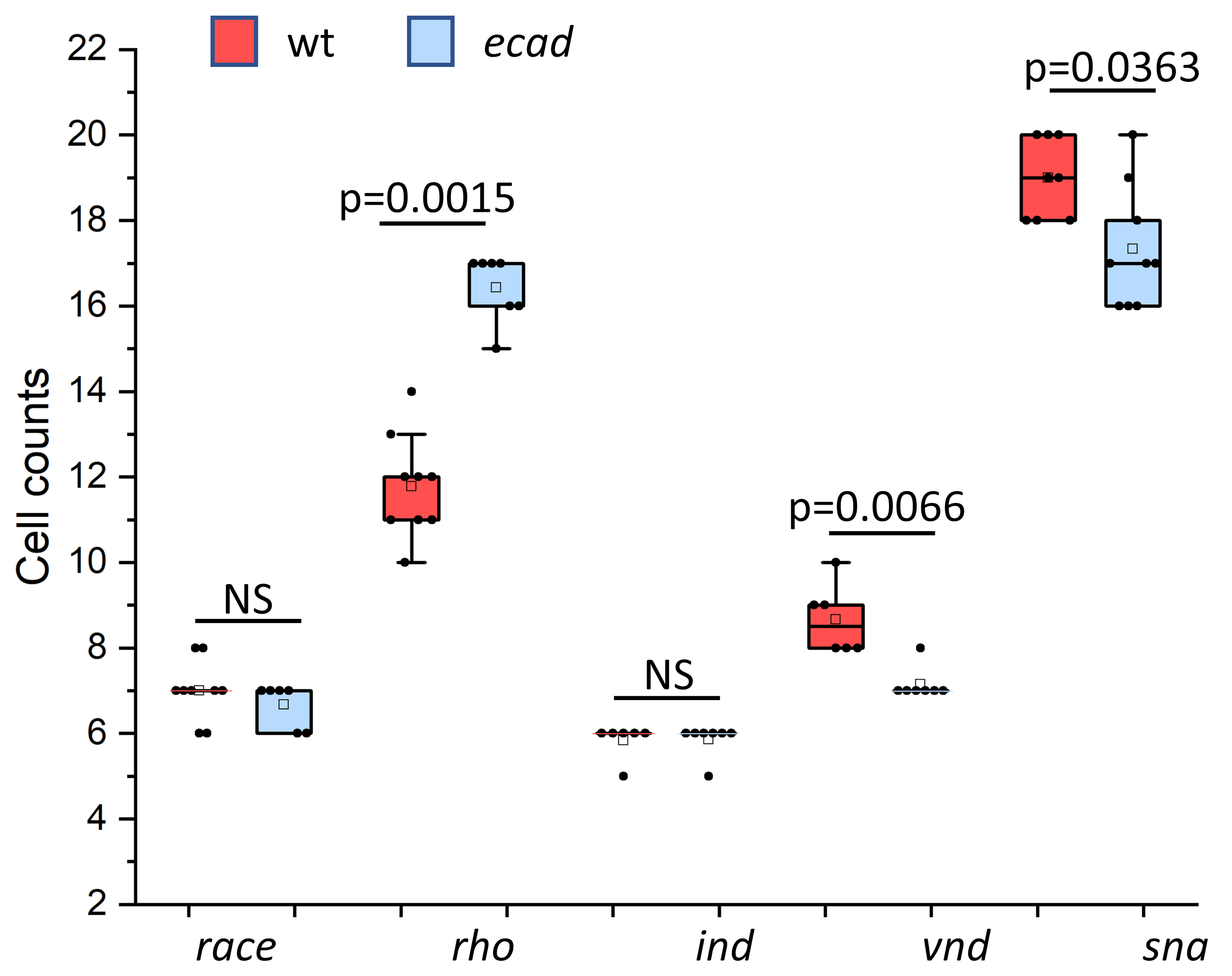

Supplement: S10 Fig — Cell counts of race and rho (ectoderm), ind and vnd (neuroectoderm), and sna (mesoderm), show that ecad mutants (blue) have enlarged rho domain and reduced vnd and sna domains compared to the wild type (red); p-values indicated on graphs were calculated with two-tail Mann–Whitney test. Metadata for the graph shown in this figure can be found at Supporting information S1 Metadata. (TIF) [file pbio.3002021.s012.TIF]
